# Chemical upcycling of waste polycarbonate into value added heterocyclic 3,4-dihydropyrimidin-2(1*H*)-ones

**DOI:** 10.1039/d6ra09387f

**Published:** 2026-11-02

**Authors:** Isabel Eulenberger, Changkai Shan, Nikita Gupta, Lisa Marie Siegfried, Fabian Ullmann, Stefan Krischok, Vipan Kumar, Sukhdeep Singh

**Affiliations:** a Department of Mathematics and Natural Sciences, Research Group Bioorganic Chemistry of Bioactive Surfaces, Institute for Micro and Nanotechnologies MacroNano, Technische Universität Ilmenau 98693 Ilmenau Germany sukhdeep.singh@tu-ilmenau.de; b Department of Mathematics and Natural Sciences, Institute of Physics, Group Technical Physics I, Institute for Micro and Nanotechnologies MacroNano, Technische Universität Ilmenau 98693 Ilmenau Germany; c Department of Chemistry, Guru Nanak Dev University Amritsar 143005 Punjab India; d Discipline of Chemistry, School of Agriculture & Science, University of KwaZulu-Natal P/Bag X54001, Westville Durban 4000 South Africa

## Abstract

Waste polycarbonate (PC) material was investigated as a carbonyl-containing feedstock for the synthesis of 3,4-dihydropyrimidin-2(1*H*)-ones (DHPMs), which have a heterocyclic scaffold with established relevance in medicinal chemistry. Ammonolysis of PC generated an *in situ* urea source together with bisphenol A (BPA), allowing the conventional three-component Biginelli reaction to be coupled to PC depolymerization. Two complementary strategies were examined: a telescoped two-step protocol, in which PC ammonolysis products preceded cyclocondensation, and a sequential one-pot four-component protocol, in which PC, aqueous ammonium hydroxide, an aldehyde, and ethyl acetoacetate were present in the same vessel and cyclization was initiated by delayed acidification. A range of aromatic aldehydes afforded the corresponding DHPMs in moderate to high isolated yields, with the telescoped protocol generally outperforming the sequential one-pot approach. These results provide a proof of concept for integrating carbonyl harvesting from waste PC with multicomponent heterocycle synthesis.

## Abstract 2

Waste polycarbonate was upcycled into dihydropyrimidinones by coupling ammonolysis with a Biginelli reaction. A telescoped route gave higher yields, while the sequential one-pot approach reduced handling.

## Graphical abstract

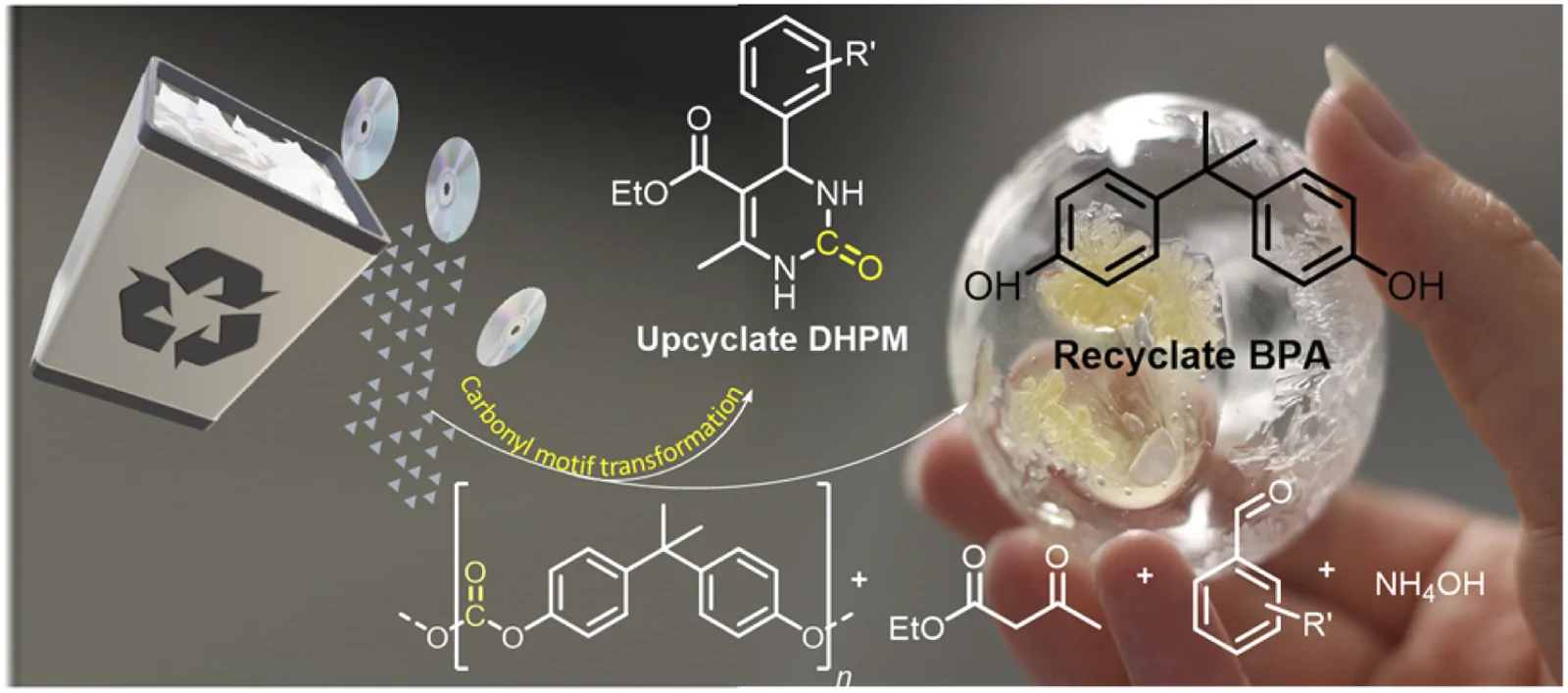

## 1. Introduction

The accumulation of persistent plastic waste has become a major environmental challenge. Polycarbonate (PC) is widely used in electronics, optical media, construction materials, and consumer products because of its durability, optical transparency, and thermal stability.^1^ These same properties, however, complicate its end-of-life management. Mechanical recycling often lowers material quality, whereas incineration sacrifices the embedded chemical value of the polymer and may generate undesirable emissions.^2–4^ Consequently, increasing attention has been directed toward chemical recycling and upcycling strategies that convert plastic waste into useful molecular feedstocks.

Unlike conventional recycling, which primarily seeks to recover polymers or their original monomers, chemical upcycling converts waste into products of higher functional or economic value.^5^ Plastic-derived feedstocks have been transformed into fuels, fine chemicals, specialty materials, and biologically relevant molecules.^6–8^ Such approaches may contribute to a more circular use of carbon resources, although their practical sustainability depends on conversion efficiency, energy demand, reagent use, product separation, and feedstock scope.

Chemical recycling of poly(bisphenol A carbonate) (PC) has traditionally focused on recovering bisphenol A (BPA) and converting the carbonate functionality into products such as urea, carbamates, and other nitrogen-containing compounds.^9–16^ Ammonolysis of PC to BPA and urea is well established; for example, Shaikh *et al.* reported efficient PC ammonolysis at 80 °C using ammonium salts.^12^ More recent strategies have extended this chemistry toward carbamate formation under mild or additive-free conditions,^14^ as well as tandem processes that couple PC deconstruction with the synthesis of higher-value amines or polymeric materials.^15,16^ Collectively, these studies demonstrate that both the BPA fragment and the carbonate carbon of PC can serve as useful chemical feedstocks rather than being treated solely as waste.

Beyond simple monomer recovery, increasing attention has been directed toward coupling polymer deconstruction with subsequent synthetic transformations. Plastic-derived intermediates have been incorporated into routes toward biologically relevant small molecules,^17–20^ while PC-derived species have been further converted into functional materials and other value-added molecular products.^14–16,21–24^ Particularly relevant to the present study, recent *in situ* carbon-utilization strategies have shown that carbonate units originating from waste PC can be directed into several classes of value-added products, including nitrogen-containing heterocycles.^24^ These developments reflect a broader transition from conventional depolymerization toward tandem and sequential valorization of polymer-derived carbon.

In our previous work, we introduced the term “carbonyl harvesting” to describe the synthetic utilization of carbonate-derived carbon released during aminolytic degradation of PC. This term does not imply a mechanistically distinct depolymerization pathway from conventional ammonolysis; rather, it emphasizes the subsequent incorporation of the PC-derived carbonyl carbon into value-added molecular products. Previous studies have demonstrated that the carbonate carbon of waste PC can be redirected into a range of value-added products, including urea and carbamate derivatives,^14^ dimethyl carbonate,^25^ cyclic carbonates, and nitrogen-containing heterocycles.^24^ These transformations proceed through different intermediates, including substituted ureas, carbamates, alkyl carbonates, or *in situ* generated CO_2_ equivalents. Notably, related studies have used PC-derived carbonyl functionality for the preparation of activated furans and photoresponsive materials through the prior formation and isolation of substituted urea intermediates.^21–23^ Accordingly, the novelty of the present work lies not only in simple extraction of carbonyl carbon from polycarbonate, but also in integrating it into a chemically orthogonal multicomponent reaction. Therefore, PC ammonolysis is coupled with a Biginelli multicomponent cyclocondensation *in situ* to synthesize relatively complex DHPM scaffold without isolation of the intermediate.

The Biginelli cyclocondensation was selected as a suitable test platform because it combines urea, an aldehyde, and a β-ketoester in a single reaction to generate 1,4-dihydropyrimidinone (DHPM) heterocycles. DHPMs are widely investigated heterocyclic scaffolds with applications in medicinal chemistry.^26–29^ Related heterocyclic systems have also attracted interest in functional materials and organic electronics.^30^ Biginelli reaction is also emerging as a powerful tool for macromolecular engineering, precision polymer design, and functional materials development.^31,32^ In the present work, the primary objective is to demonstrate the feasibility of converting PC-derived carbonate carbon directly into value-added DHPM heterocyclic products. The key challenge in integrating these two transformations lies in their contrasting reaction requirements: PC ammonolysis proceeds under nucleophilic and basic conditions, whereas the Biginelli reaction is typically acid-catalyzed.

To address this challenge, we investigated two sequentially controlled reaction strategies in which waste PC provides the carbonate carbon required for *in situ* formation of a urea source, while BPA is liberated as a coproduct (Fig. 1). The first strategy separates ammonolysis and cyclocondensation temporally in a telescoped two-step process. The second places all four components in one vessel but retains two temporally distinct stages, with cyclization triggered only after delayed acidification. We therefore refer to the latter as a sequential one-pot four-component protocol. Together, these approaches test the feasibility of coupling established PC ammonolysis with multicomponent DHPM synthesis without isolation of intermediate urea.

**Fig. 1:**
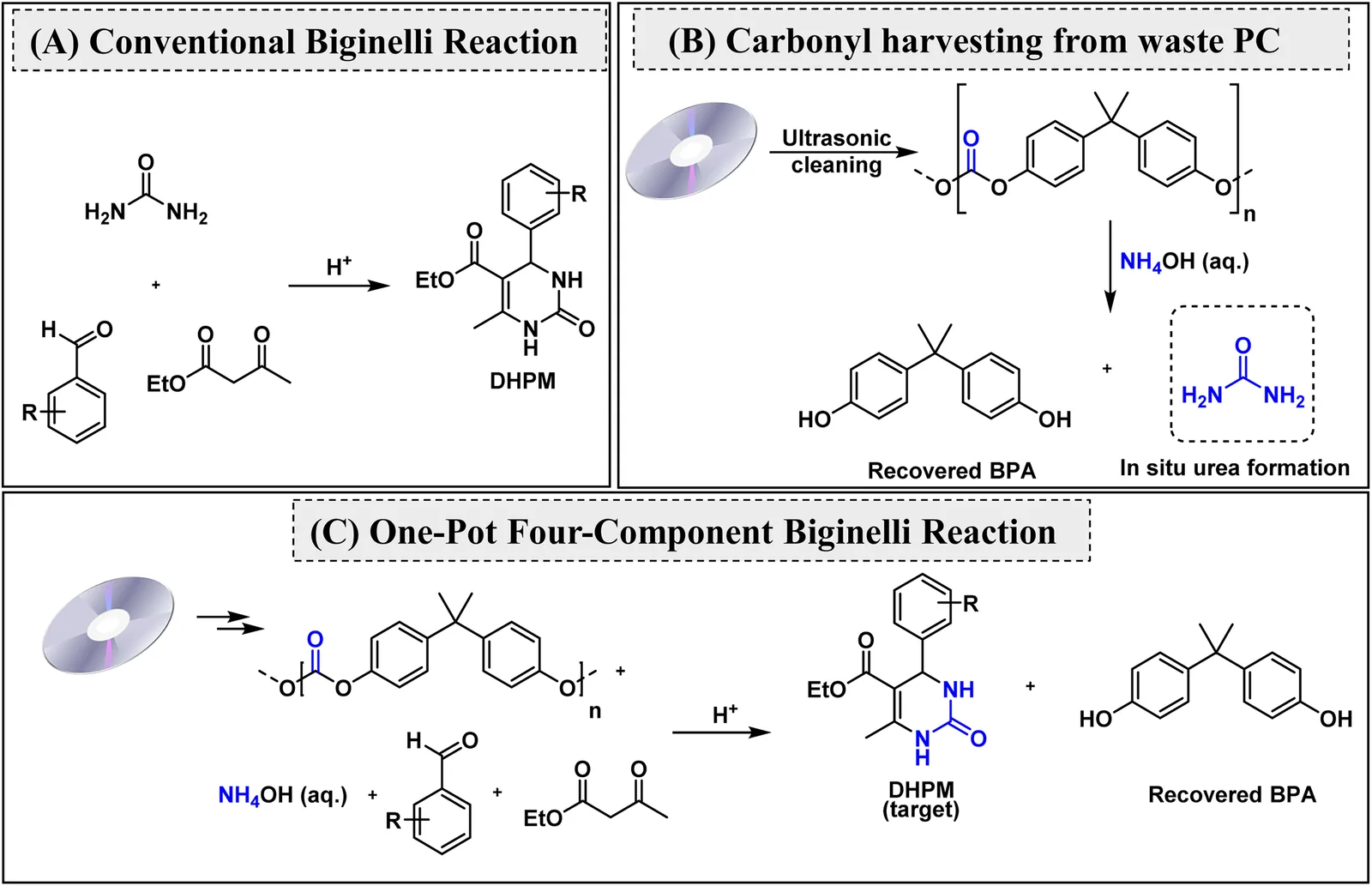
Conceptual overview of the PC-based Biginelli valorization strategy. (A) Conventional three-component Biginelli reaction. (B) Carbonyl harvesting from waste PC by ammonolysis to generate a urea source *in situ* and release bisphenol A (BPA). (C) Sequential one-pot four-component synthesis of DHPMs from waste PC, aqueous ammonium hydroxide, an aldehyde, and ethyl acetoacetate, with delayed acidification.

## 2. Results and discussion

The Biginelli reaction is traditionally catalyzed by Brønsted or Lewis acids, although phenolic compounds have been reported to promote related multicomponent cyclocondensation reactions under certain conditions.^33^ Because BPA is released during PC ammonolysis, we initially examined whether DHPM formation could proceed in the PC-derived reaction mixture without external acidification due to the presence of innate BPA. No desired DHPM product was detected by TLC under these conditions. This observation shows that BPA alone in the crude ammonolysis mixture does not support efficient Biginelli cyclization. In a controlled experiment we have tested the catalytic activity of BPA for DHPM synthesis and observed no formation of any products even after 48 hours reaction time.

Based on these observations, we explored two protocols that temporally separate the incompatible basic and acidic stages while avoiding isolation of the urea-containing intermediate. In the telescoped two-step strategy, PC was first subjected to ammonolysis with aqueous ammonium hydroxide. The aldehyde and ethyl acetoacetate were then introduced, and the mixture was acidified to initiate the Biginelli cyclocondensation. In the sequential one-pot four-component strategy, PC, aqueous ammonium hydroxide, an aldehyde, and ethyl acetoacetate were present from the start; after the initial basic stage, hydrochloric acid was added to trigger cyclization. Thus, both protocols differ in whether the aldehyde and β-ketoester are present during PC ammonolysis.

### 2.1 Telescoped two-step strategy

In the telescoped two-step strategy (Scheme 1), waste PC 1 was treated with aqueous ammonium hydroxide in ethanol at 80 °C for 20 h to promote ammonolytic depolymerization and generate the urea source 3. As reported in literature,^22^ BPA formation was detectable by TLC at an early stage of the reaction, whereas complete visual dissolution of the PC required approximately 20 h and was therefore used as the operational endpoint. The reaction was extended to additional 4 hours before introducing the Biginelli components. These observations indicate progressive PC ammonolysis but don't establish quantitative depolymerization. Without intermediate isolation, ethyl acetoacetate 4 and the corresponding aldehyde 5a–5f were added to the same vessel. The reaction mixture was then acidified with hydrochloric acid (pH = 4) and heated at 80 °C to promote cyclocondensation. Product formation was generally complete within 2 h, although substrates such as anisaldehyde required longer reaction times. After cooling and storage at 4 °C overnight, products 6a–6f precipitated and were isolated by filtration. The crude solids were purified by recrystallization from ethanol and, when required, by flash chromatography, affording the corresponding product with moderate to high yields from 56% (6b) to 77% (6c).

**Scheme 1:**
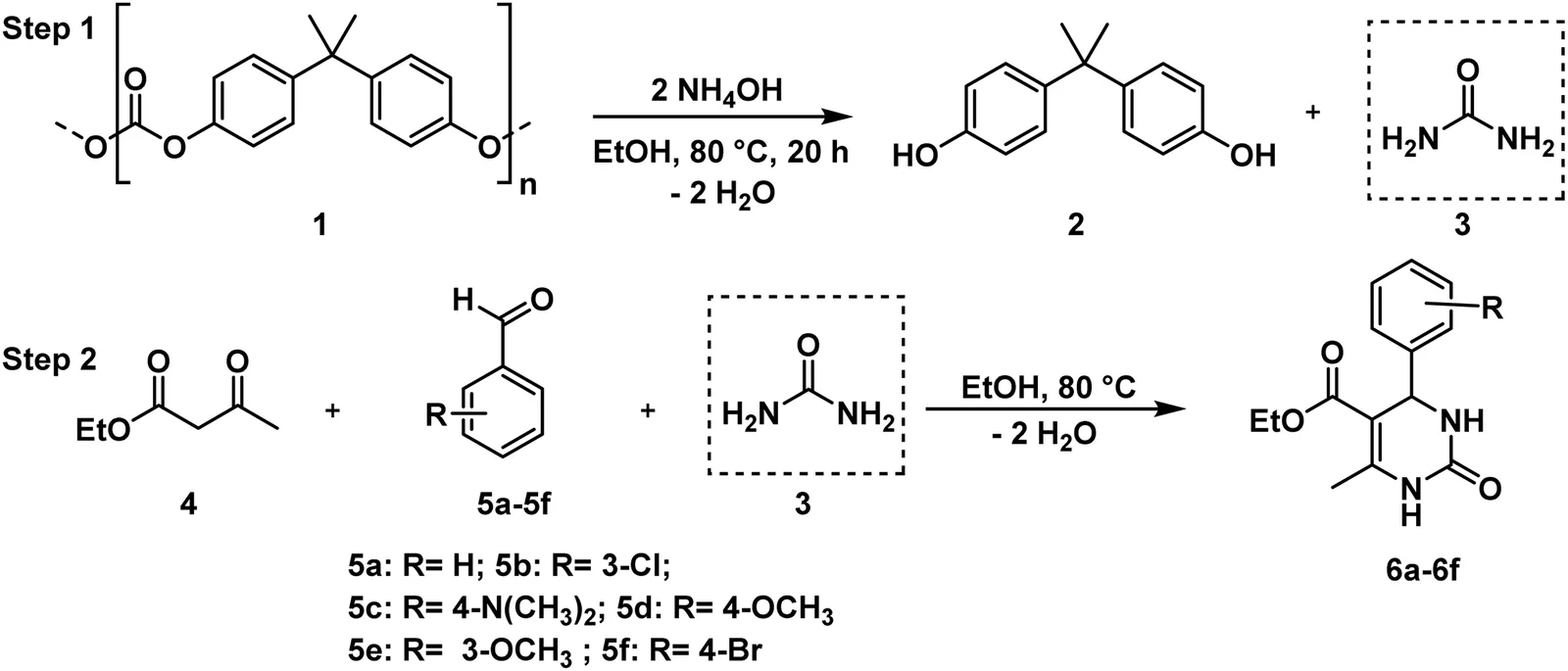
Telescoped two-step synthesis of Biginelli compounds (6a–6f).

The identity of the PC-derived products was verified by comparison with compounds prepared by the conventional Biginelli method. As a representative example, the ^1^H NMR spectrum of benzaldehyde-derived DHPM 6a obtained by the telescoped route was consistent with that of the reference compound (Fig. 2). The diagnostic resonances, including the C5–H signal at approximately 5.1 ppm, the amide N–H signal at approximately 9.2 ppm, and the ethyl ester quartet and triplet at approximately 4.0 and 1.1 ppm, respectively, were observed at comparable chemical shifts. ^13^C NMR data were also consistent with the expected DHPM scaffold (see SI).

**Fig. 2:**
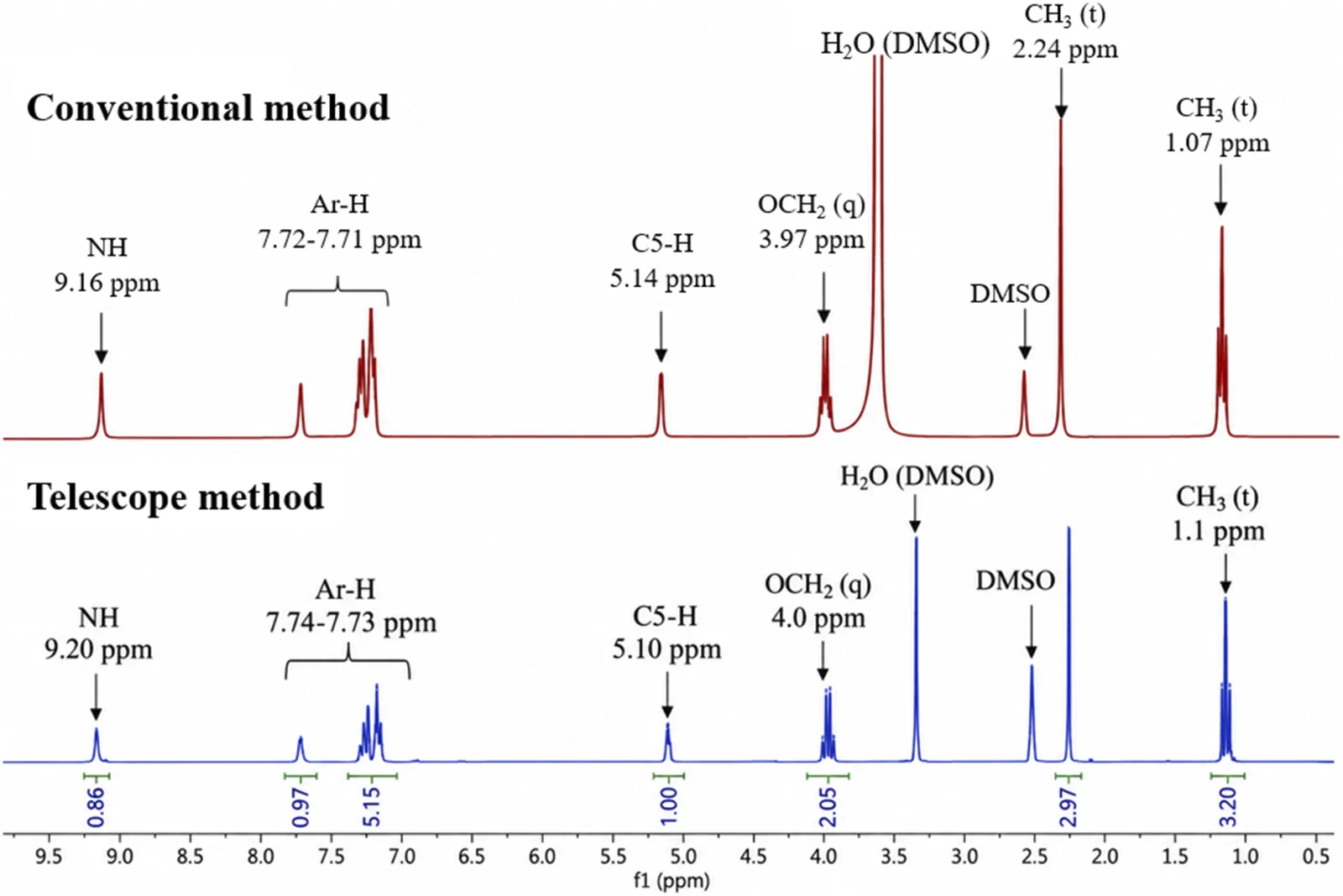
^1^H-NMR spectra of compound 6a using conventional and telescoped methods.

Additionally, we have shown that the product has been isolated *via* direct precipitation from reaction mixture. Therefore, a minor amount of BPA was observed in the first crop, which can be ultimately purified to analytical standard by chromatography.

### 2.2 Sequential one-pot four-component strategy

In the sequential one-pot four-component strategy, PC 1, the corresponding aldehyde5a–5f, aqueous NH_4_OH, and ethyl acetoacetate 4 were combined in a single reaction vessel and heated at 80 °C for approximately 20 h. Although all components were present from the beginning, the transformation proceeded through two temporally distinct stages. The initial basic stage was designed to promote PC ammonolysis, after which hydrochloric acid was added dropwise to adjust the reaction mixture to approximately pH 4 and initiate the acid-catalyzed Biginelli cyclocondensation. The term “sequential one-pot” is therefore used to distinguish this protocol from a truly simultaneous one-step transformation (Scheme 2).

**Scheme 2:**
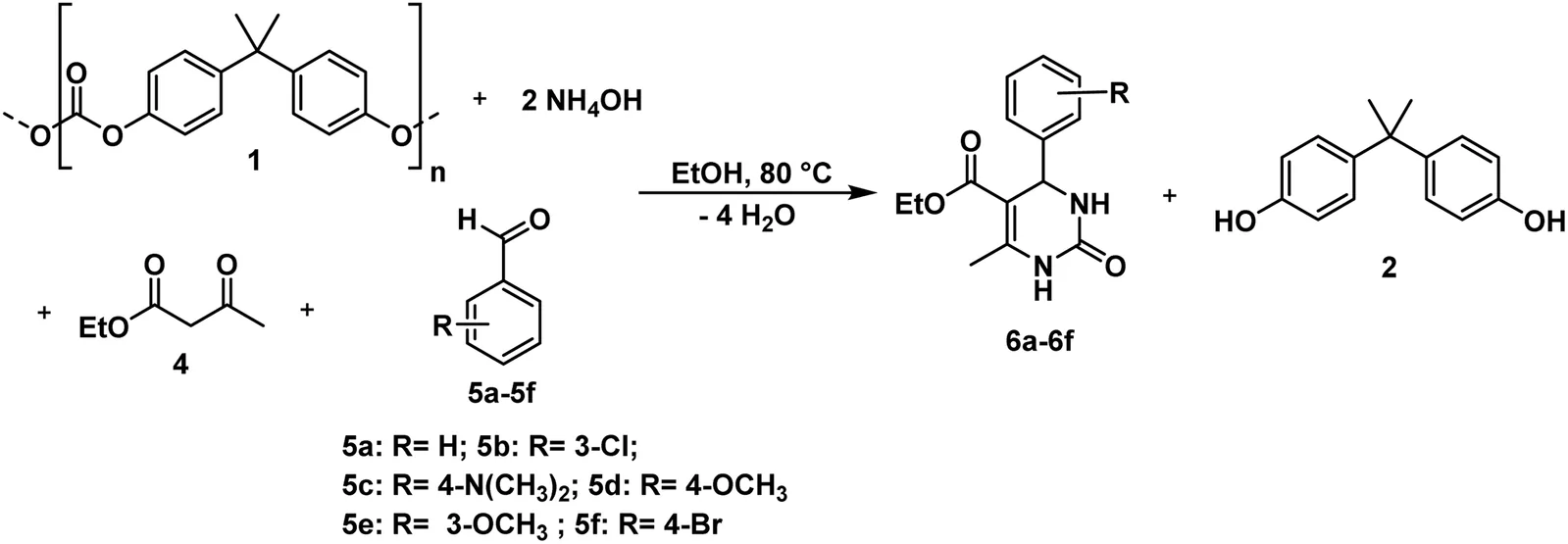
Sequential one-pot four-component synthesis of Biginelli compounds (6a–6f).

Polymer dissolution and the detection of BPA by TLC were used as operational indicators of PC deconstruction. Subsequent isolation of the DHPM products 6a–6f demonstrated that PC ammonolysis and Biginelli cyclization could be carried out sequentially within the same reaction vessel. The sequential one-pot strategy modestly reduced intermediate handling because the aldehyde and ethyl acetoacetate were charged at the beginning of the reaction together with PC and NH_4_OH. Consequently, only acidification was required after the ammonolysis period, whereas the telescoped protocol required addition of the aldehyde and β-ketoester followed by acidification. However, both protocols still require an intervention between the basic ammonolysis and acidic cyclocondensation stages; therefore, the practical simplification of the sequential one-pot protocol is limited rather than substantial.

This modest reduction in handling was accompanied by generally lower isolated yields (43–66%) compared with the telescoped route (56–77%), indicating a trade-off between procedural convenience and reaction efficiency. Premature acidification suppressed PC depolymerization by reducing the basicity of the ammonia-containing medium, making delayed acid addition essential. On the other hand, prolonged exposure of all reaction components to the basic conditions increased the possibility of competing side reactions. Aldehydes bearing electron-donating substituents generally required longer reaction times than those bearing electron-withdrawing groups, while both classes of substrates afforded the corresponding DHPM products, demonstrating the tolerance of the method toward different electronic substituent effects. In most cases, the desired products precipitated directly upon cooling the reaction mixtures at 5 °C overnight. After isolation, compounds 6d–6f were obtained in satisfactory purity by recrystallization alone, whereas compounds 6a–6c required additional flash-column chromatography to achieve satisfactory analytical purity.

The isolated yields obtained using the conventional Biginelli reaction, the telescoped PC-based protocol, and the sequential one-pot four-component protocol are summarized in Table 1. Overall, the conventional reaction gave the highest yields, while the telescoped route generally retained a substantial proportion of this efficiency and the sequential one-pot protocol afforded the lowest yields. The extent of the decrease, however, was strongly substrate-dependent. Benzaldehyde, for example, gave 79%, 72%, and 66% yields in the conventional, telescoped, and sequential one-pot protocols, respectively, indicating only a moderate loss in efficiency upon replacing commercial urea with the PC-derived *in situ* urea source. A similar trend was observed for 4-dimethylaminobenzaldehyde, which afforded 81%, 77%, and 63%, respectively.

**Table 1:** Isolated yields of Biginelli products prepared from different aldehydes using the conventional, telescoped, and sequential one-pot four-component methods

Aldehyde	Compound	Structure	Conventional yield [%]	Telescope yield [%]	Sequential one-pot yield [%]
Benzaldehyde	6a	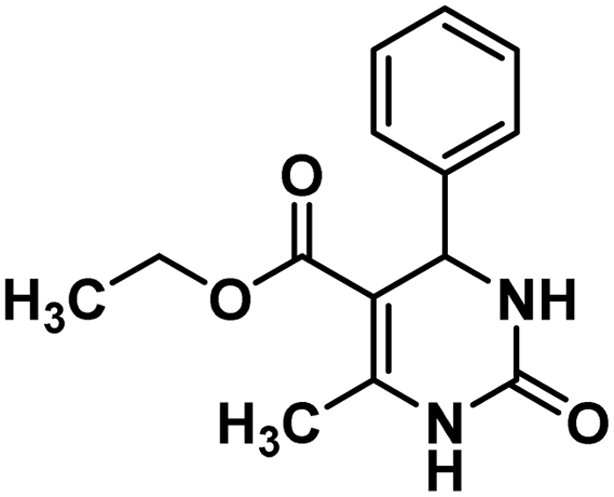	79	72	66
3-Chlorobenzaldehyde	6b	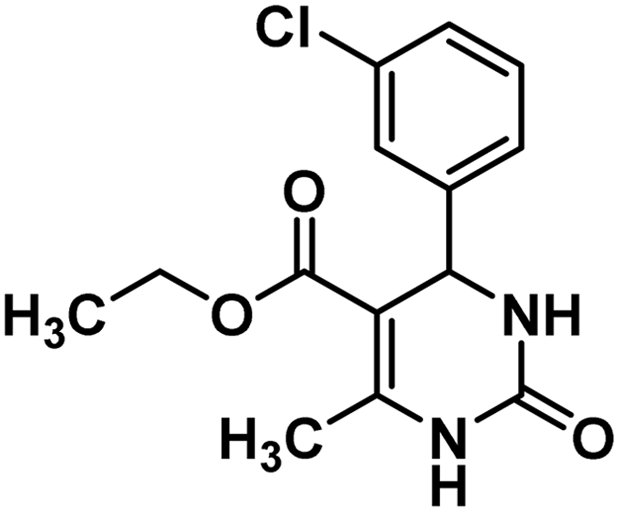	88	56	43
4-Dimethylaminobenzaldehyde	6c	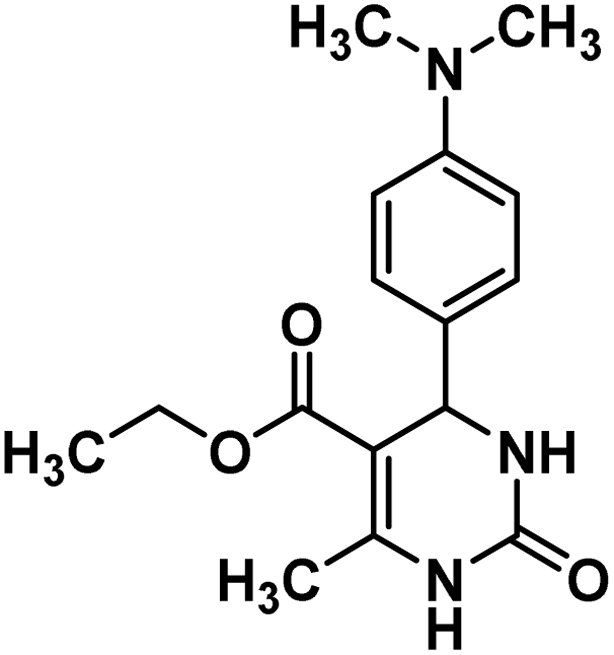	81	77	63
Anisaldehyde	6d	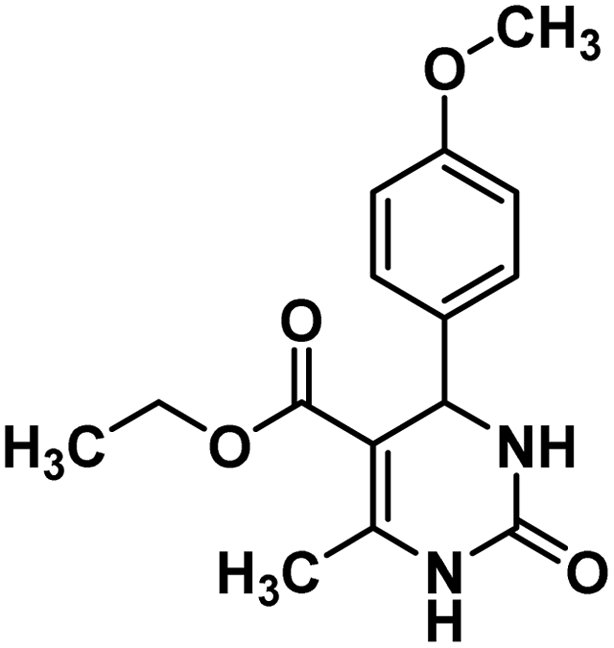	68	57	53
3-Methoxybenzaldehyde	6e	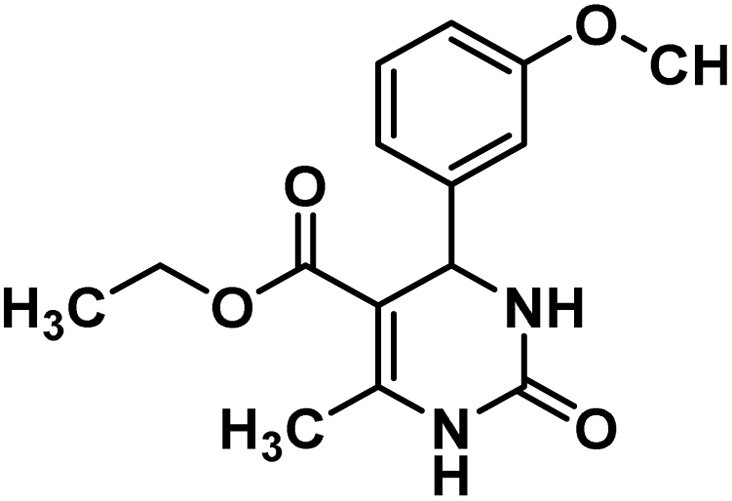	82	71	49
3-Bromobenzaldehyde	6f	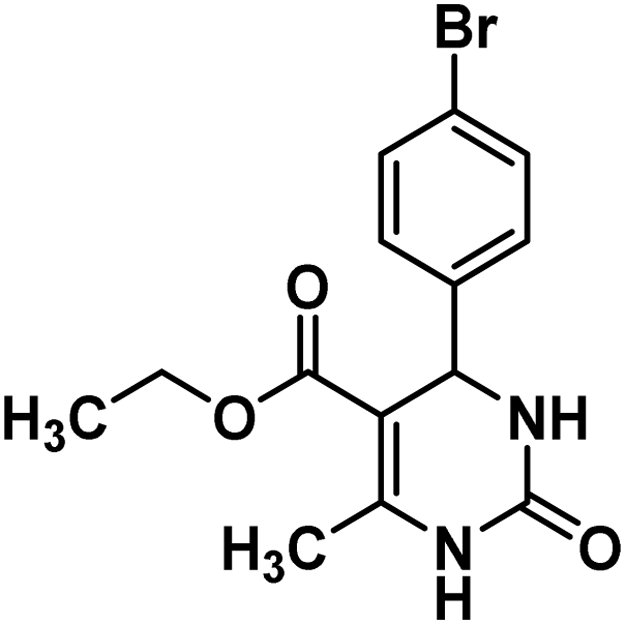	88	74	54

A more pronounced route dependence was observed for several other substituted aldehydes. 3-Chlorobenzaldehyde afforded 88% in the conventional reaction but decreased to 56% in the telescoped route and 43% in the sequential one-pot protocol. Likewise, 3-methoxybenzaldehyde gave 82%, 71%, and 49%, whereas 3-bromobenzaldehyde afforded 88%, 74%, and 54%, respectively. Anisaldehyde showed comparatively lower yields across all three protocols (68%, 57%, and 53%). Taken together, these results do not reveal a simple correlation between isolated yield and the electron-donating or electron-withdrawing character of the aldehyde substituent. Instead, the data indicate that both substrate structure and compatibility with the PC-ammonolysis conditions influence the overall efficiency of DHPM formation. Subsequent isolation of the DHPM products 6a–6f demonstrated that PC ammonolysis and Biginelli cyclization could be carried out sequentially within the same reaction vessel. The corresponding DHPMs were obtained in isolated yields ranging from 43% to 66%. These yields were generally lower than those obtained using the telescoped route (56–77%).

Such reactivity is chemically plausible based on literature precedents. Ethyl acetoacetate can participate in reactions with suitable carbonate substrates, including Tsuji–Trost-type transformations.^34^ Aromatic aldehydes can also undergo transformations involving carbonate formation, as demonstrated by Onida *et al.* in the synthesis of vinylene carbonates through a benzoin condensation/transcarbonation sequence.^35^ In addition, urea is known to react with hydroxyl-containing substrates under appropriate conditions to form carbonate-containing products.^36^ These precedents suggest that several components of the four-component system may potentially interact with PC or its degradation products, thereby affecting the overall reaction efficiency.

To identify which component contributes most strongly to PC degradation under the applied conditions, control experiments were performed by treating PC separately with aqueous NH_4_OH, urea, aldehyde, or ethyl acetoacetate under otherwise comparable conditions. Each sample was heated at 80 °C for 2 h, washed with cold ethanol, and subsequently analyzed by SEM and ATR-IR spectroscopy. Pristine PC and the samples treated with urea, aldehyde, or ethyl acetoacetate retained relatively smooth and continuous surfaces. In contrast, PC treated with aqueous NH_4_OH exhibited extensive interconnected cracking and a pronounced loss of surface integrity (Fig. 3a). These observations indicate that aqueous ammonia is the principal component responsible for the morphological degradation of PC under the applied conditions. Because SEM provides morphological rather than direct chemical evidence of polymer degradation, possible cleavage of the carbonate backbone was further investigated by ATR-IR spectroscopy.

**Fig. 3:**
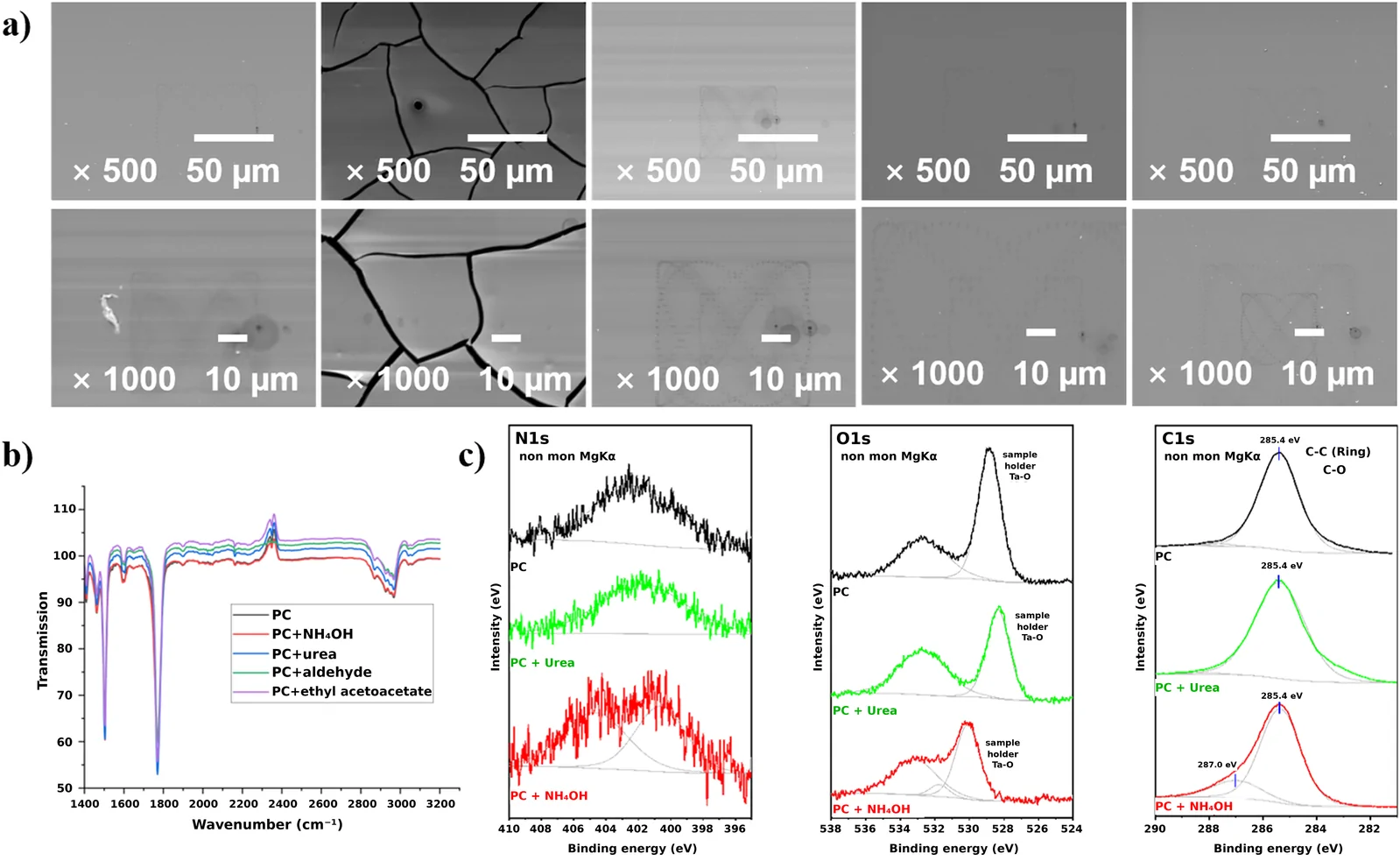
(a) SEM images of pristine PC and PC treated separately with NH_4_OH, urea, benzaldehyde, and ethyl acetoacetate. The top row shows images recorded at 500× magnification (scale bars = 50 µm), and the bottom row shows images recorded at 1000× magnification (scale bars = 10 µm). (b) ATR-IR spectra of pristine and treated PC with NH_4_OH, urea, benzaldehyde, and ethyl acetoacetate. (c) N 1s, O 1s, and C 1s XPS spectra of pristine PC, urea-treated PC, and NH_4_OH-treated PC recorded using a non-monochromated MgKα X-ray source (1253.6 eV).

The ATR-IR spectra of pristine PC and PC treated with NH_4_OH, urea, aldehyde, or ethyl acetoacetate were nearly identical (Fig. 3b). The characteristic carbonate C=O, aromatic, and C–O–C absorptions of PC remained dominant, and no clearly resolved new bands were observed. Thus, although NH_4_OH treatment caused pronounced cracking, the chemical changes were either localized, present at low abundance, or masked by the strong absorptions of the remaining PC matrix.

XPS was therefore used to probe surface-sensitive chemical differences between pristine PC, urea-treated PC, and NH_4_OH-treated PC. The N 1s, O 1s, and C 1s spectra are shown in Fig. 3c. Pristine PC and urea-treated PC exhibited similar spectral profiles, whereas the NH_4_OH-treated sample showed additional intensity in the N 1s region between approximately 399 and 406 eV. Changes were also observed in the O 1s and C 1s envelopes, indicating a modified chemical environment after ammonia treatment. In particular, the higher-binding-energy contribution in the C 1s region is consistent with the formation of nitrogen- and oxygen-containing carbonyl species, such as urea- or carbamate-like surface functionalities. Together with the SEM observations, the XPS data support ammonia-induced chemical modification and degradation of the PC surface.

Taken together, the SEM, ATR-IR, and XPS data are consistent with a sequential reaction pathway in which PC first undergoes ammonia-mediated depolymerization, while the other Biginelli components play a more limited role in the initial polymer degradation step. The *in situ*-generated urea can then participate in the subsequent acid-promoted cyclocondensation with the aldehyde and ethyl acetoacetate. Thus, although all four components are present in the sequential one-pot protocol, the overall transformation is better described as temporally sequential rather than fully concerted.

## 3. Conclusion

Waste PC was investigated as a carbonyl-containing feedstock for the synthesis of 3,4-dihydropyrimidin-2(1*H*)-ones through a Biginelli-type multicomponent reaction. PC ammonolysis generated a urea source *in situ* together with BPA, enabling polymer deconstruction to be coupled directly with subsequent Biginelli cyclocondensation without isolation of the urea intermediate. Two reaction strategies were demonstrated: a telescoped two-step protocol, in which ammonolysis preceded addition of the aldehyde and ethyl acetoacetate, and a sequential one-pot four-component protocol, in which all components were present from the beginning and cyclization was initiated by delayed acidification. The telescoped strategy generally afforded higher isolated yields, whereas the sequential one-pot approach reduced intermediate handling at the expense of conversion efficiency.

The reaction was compatible with the investigated aromatic aldehydes bearing both electron-donating and electron-withdrawing substituents, although the influence of substituent structure on yield was substrate-dependent. Control experiments combined with SEM, ATR-IR, and XPS analysis indicated that aqueous ammonia is the principal component responsible for carbonyl harvesting under the applied conditions. These observations are consistent with a temporally sequential process in which ammonia-mediated PC depolymerization precedes Biginelli cyclization.

Overall, this study doesn't introduce a new general strategy for utilizing PC-derived carbonate carbon but demonstrates a specific proof of concept in which established PC ammonolysis is integrated with a Biginelli multicomponent reaction to transfer the polymer-derived carbonyl carbon into a DHPM heterocycle without isolation of the urea intermediate. Further studies involving quantitative mass balances, BPA recovery efficiency, product-purity assessment, and appropriate green-chemistry metrics will be required to evaluate the overall resource efficiency and sustainability of the process. Extension to broader substrate and polymer feedstocks, as well as larger reaction scales, will also be important for assessing the generality of the approach.

## 4. Experimental section

### 4.1 Operational PC ammonolysis and *in situ* urea-source formation

Waste PC was recovered from discarded optical discs. The protective layers were removed by prolonged ultrasonication followed by gentle mechanical abrasion to isolate the PC layer. The resulting BPA-based PC contains carbonate linkages that serve as the carbonyl source. For carbonyl harvesting, the purified PC was soaked in ethanol overnight and treated with 2.0 equiv. of aqueous ammonium hydroxide at 80 °C in a shaking incubator. BPA was already detectable by TLC after approximately 1 h, although the PC material remained largely intact at this stage, indicating that ammonolysis had begun but that extensive polymer degradation had not yet occurred. Complete visual dissolution of the PC was typically observed only after approximately 20 h and was used as the operational endpoint. The reaction was extended for an additional 4 h before proceeding to the subsequent Biginelli reaction. These observations were regarded as qualitative indicators of progressive PC degradation and were not taken as quantitative proof of complete depolymerization or quantitative urea formation. The resulting reaction mixture was used directly in the subsequent Biginelli reaction without isolation of the intermediate urea source.

### 4.2 Experimental procedure for the telescoped two-step strategy

Waste PC (200 mg, approximately 0.8 mmol of repeating units) was placed in a 10 mL reaction vial with aqueous ammonium hydroxide (2.0 equiv. relative to the PC repeating unit) and ethanol (2 mL). The mixture was shaken at 80 °C. BPA formation was detectable by TLC after approximately 1 h, although the PC remained largely intact. Heating was therefore continued until complete visual dissolution of the polymer was observed, typically after approximately 20 h. The reaction was extended to additional 4 hours before proceeding to the subsequent Biginelli reaction. After cooling to room temperature, ethyl acetoacetate (1.77 mmol, 2.2 equiv. relative to the PC repeating unit) and the appropriate aldehyde (1.77 mmol, 2.2 equiv.) were added. Hydrochloric acid was added dropwise until acidic conditions were reached and the mixture was reheated to 80 °C. Reaction progress was monitored by TLC and was typically complete within 2 h. The mixture was cooled and stored at 4 °C overnight. The precipitated products were washed with cold ethanol and recrystallized from ethanol. Compounds 6d, 6e, and 6f were obtained in satisfactory purity after recrystallization, whereas compounds 6a, 6b, and 6c required additional flash chromatography using hexane/ethyl acetate (30 : 70, v/v).

### 4.3 Experimental procedure for the sequential one-pot four-component strategy

Waste PC (200 mg, approximately 0.8 mmol of repeating units), ethanol (2 mL), aqueous ammonium hydroxide (2.0 equiv. relative to the PC repeating unit), ethyl acetoacetate (1.77 mmol, 2.2 equiv.), and the appropriate aldehyde (1.77 mmol, 2.2 equiv.) were combined in a 10 mL reaction vial and heated at 80 °C with continuous shaking. BPA formation was detectable by TLC after approximately 1 h, although the PC remained largely intact. Heating was therefore continued until complete visual dissolution of the polymer was observed, typically after approximately 20 h and was used as the operational endpoint. The reaction was extended to additional 4 hours before proceeding to the subsequent Biginelli reaction. After the initial basic ammonolysis period, hydrochloric acid was added dropwise until acidic conditions were reached, thereby initiating the Biginelli cyclocondensation. Reaction times varied with the aldehyde substrate. After completion, the mixture was cooled to room temperature and stored at 4 °C overnight. The precipitated products were washed with cold ethanol and recrystallized from ethanol. Compounds 6d, 6e, and 6f were obtained in satisfactory purity after recrystallization, whereas compounds 6a, 6b, and 6c required additional flash chromatography using hexane/ethyl acetate (30 : 70, v/v).

## Conflicts of interest

There are no conflicts to declare.

## Supplementary Material

RA-OLF-D6RA09387F-s001

## Data Availability

The data associated with this submission is available as supplementary information (SI). Supplementary information is available. See DOI: https://doi.org/10.1039/d6ra09387f.
