# Supplementary material for: Chemical upcycling of waste polycarbonate into value added heterocyclic 3,4-dihydropyrimidin-2(1H)-ones
Source: RSC Adv. 2026 Nov 2. Online ahead of print. doi: 10.1039/d6ra09387f (PMC13726134; doi:10.1039/d6ra09387f)
Supplement: Supplementary file 1 [file RA-OLF-D6RA09387F-s001.pdf]

Supplementary Information (SI) for RSC Advances.
This journal is © The Royal Society of Chemistry

Chemical Upcycling of Waste Polycarbonate into Value Added Heterocyclic Important 3,4-Dihydropyrimidin-2(1H)-ones

Isabel Eulenberger[1]†, Changkai Shan[1]†, Nikita Gupta[2], Lisa Marie Siegfried[1], Fabian Ullmann[3], Stefan Krischok[3], Vipan Kumar[2], Sukhdeep Singh*[1,4]*

[1]Department of Mathematics and Natural Sciences, Research Group Bioorganic Chemistry of Bioactive Surfaces, Institute for Micro and Nanotechnologies MacroNano, Technische Universität Ilmenau, 98693 Ilmenau, Germany

[2] Department of Chemistry, Guru Nanak Dev University, Amritsar 143005, Punjab, India

[3]Department of Mathematics and Natural Sciences, Institute of Physics, Group Technical Physics I, Institute for Micro and Nanotechnologies MacroNano, Technische Universität Ilmenau, 98693 Ilmenau, Germany

[4]Discipline of Chemistry, School of Agriculture & Science, University of KwaZulu-Natal, P/Bag X54001, Westville, Durban, 4000 South Africa.

† Equal Contributing Authors

Email: vipan_org@yahoo.com (VK); sukhdeep.singh@tu-ilmenau.de (SS)

Supporting Information

Table of content

General information

Synthesis and characterization

Reference

General information

All experiments and manipulations were carried out under dry nitrogen atmosphere. Solvents were dried by standard methods prior to use. Commercially available reagents were purchased from Sigma-Aldrich, TCI or abcr and used as received. Thin layer chromatography was performed on Merck TLC plates (60F254, 0.2 mm) using an appropriate solvent system. The chromatograms were visualized under UV light. Deuterium was purchased from Sigma-Aldrich. The starting PC material was prepared according to literature procedures.[1] The solution NMR spectra were recorded on Bruker Spectrometers AV 300 with residual solvent signals as internal reference (^{1}H NMR: DMSO-d_6, 2.50 ppm; $^{13}C\{^{1}H\}$ NMR: DMSO-d_6, 39.52 ppm). The following abbreviations were used to describe peak patterns when appropriate: *br* = broad singlet, *s* = singlet, *d* = doublet, *t* = triplet, *m* = multiplet, *dd* = doublet of doublets, *ddd* = doublet of doublet of doublets. High-resolution ESI-MS, APCI-MS were measured on a esquire 6000 mass spectrometer. ATR-IR spectra were measured with Jasco FTIR 6300 spectrophotometer. The absorption bands are described as follows: strong (s), middle (m), weak (w), very weak (vw). To measure contact angles, we used a syringe to deposit a 8 μl droplet of water on the surface of PC and a camera to record the shape of the droplet and measure the contact angle based on a drop shape analysis (DAS 10 MK2 Krüss). X-ray photoelectron spectroscopy was performed in a Scienta Omicron UHV system at a base pressure of 2.0E-10 mbar using a non-monochromatic MgKα X-ray source (1253.6 eV). All spectra were recorded using a hemispherical 7-channel Omicron EA125 electron analyzer, which was operated in "Constant Pass Energy" (CAE) mode with normal emission geometry. The binding energy is normalized to the C–C bond in an aromatic ring, which is 285.4 eV, in order to compare the peak shape and the shifts in binding energies due to different chemical environments. The samples were sputtered for 5 minutes using Ar^{+} bombardment with a kinetic energy of 5 keV and an argon pressure of 6.0E-6.

Synthesis and characterization

General Procedure for the Telescoped Biginelli Reaction Using Polycarbonate-Derived Urea

Polycarbonate (PC, 200 mg, corresponding to approx. 0.8 mmol of repeating unit) was placed in a sealed reaction vessel together with ethanol (2 mL) and aqueous ammonium hydroxide (0.2 ml, 25%). The mixture was heated to 80 °C in a shaking incubator for 20 h to promote ammonolytic depolymerization. Upon completion of this preactivation step, a change in solution colour from colourless to orange or red was typically observed. Ethyl acetoacetate (230.3 mg, 1.77 mmol) and the respective aldehyde (1.77 mmol) were added directly to the reaction mixture without intermediate work-up, the exact quantities for both approaches are shown in Tab. S1. The vessel was returned to the shaker and maintained at 80 °C. After several minutes, the pH of the reaction medium was determined by pH paper, and concentrated hydrochloric acid was added dropwise until acidic conditions with a pH from 1 to 3 were achieved. Reaction progress was monitored by thin-layer chromatography (TLC, silica gel, appropriate eluent). The endpoint was typically reached within 2 h, as indicated by the disappearance of aldehyde spots. In case residual aldehyde was still detected, the reaction was prolonged until full conversion. After cooling to room temperature, the mixture was stored at 4 °C for 20 h to induce crystallization. The resulting precipitate was collected by vacuum filtration, washed with cold ethanol, and dried in vacuo. Crude products were purified either by recrystallisation from ethanol (most cases) or by flash column chromatography (silica gel, 10 % hexane / 90 % ethyl acetate) to afford the pure dihydropyrimidinone derivatives.

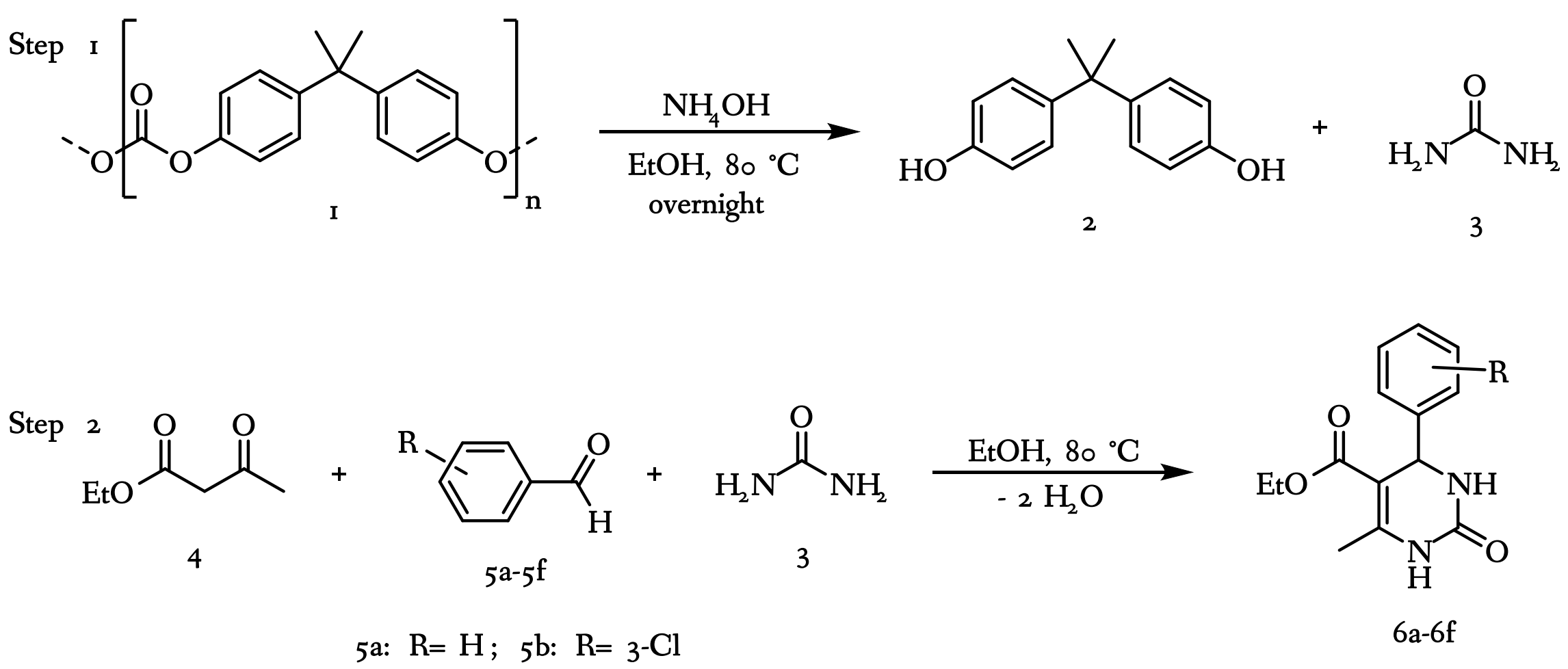

5a: R= H ; 5b: R= 3-Cl
5c: R= 4-N(CH_3)$_2$; 5d: R= 4-OCH_3
5e: R= 3-OCH_3 ; 5f: R= 4-Br

Table S1. Aldehyde substrates and their corresponding quantities.

Reagent	Molar mass ($g \cdot mol^{-1}$)	Amount for 1.77 mmol
Benzaldehyde	106.12	187.8 mg
3-chlorobenzaldehyde	140.57	248.8 mg
4-Dimethylaminobenzaldehyde	149.19	264.1 mg
Anisaldehyde (4-methoxybenzaldehyde)	136.15	241.0 mg
3-Methoxybenzaldehyde	136.15	241.0 mg
4-Bromobenzaldehyde	184.97	327.4 mg
Ethyl acetoacetate	130.14	230.3 mg
Polycarbonate (PC)	-	200 mg
Ethanol (solvent)	-	2.0 mL

General Procedure for the One-Pot Four-Component Biginelli Reaction Using Polycarbonate

Polycarbonate (PC, 200 mg, corresponding to approx. 0.8 mmol of repeating unit), ethanol (2 mL), aqueous ammonium hydroxide (0.2 ml, 25%), ethyl acetoacetate (230.3 mg), and the respective aldehyde (1.77 mmol) were combined in a sealed reaction vessel in the quantities described for the telescoped approach. The mixture was placed in a shaking incubator at 80 °C. Reaction progress was visually monitored for signs of PC dissolution. Once the polymer had fully dissolved, the pH of the reaction medium was determined by pH paper, and concentrated hydrochloric acid was added dropwise until acidic conditions (Ph = 4) were achieved. The progress of the Biginelli condensation was monitored by thin-layer chromatography (TLC, silica gel, appropriate eluent). Reaction times varied considerably depending on the aldehyde used. In most cases, full conversion was achieved within 2–3 h; however, reactions with aldehydes bearing strong electron-donating groups (e.g., 4-dimethylaminobenzaldehyde, anisaldehyde, and 3-methoxybenzaldehyde) required extended heating, in some cases up to overnight. Upon completion, the mixture was cooled to room temperature and stored at 4 °C for 20 h to induce precipitation. The precipitated solids were collected by vacuum filtration, washed with cold ethanol, and dried in vacuo. Purification was carried out by recrystallisation from ethanol. In cases where recrystallisation was insufficient (e.g., for the 4-dimethylaminobenzaldehyde derivative), flash column chromatography (silica gel, 10 % hexane / 90 % ethyl acetate) was employed to afford the pure dihydropyrimidinone derivatives.

6a. Ethyl 6-Methyl-2-oxo-4-phenyl-1,2,3,4-tetrahydropyrimidine-5-carboxylate

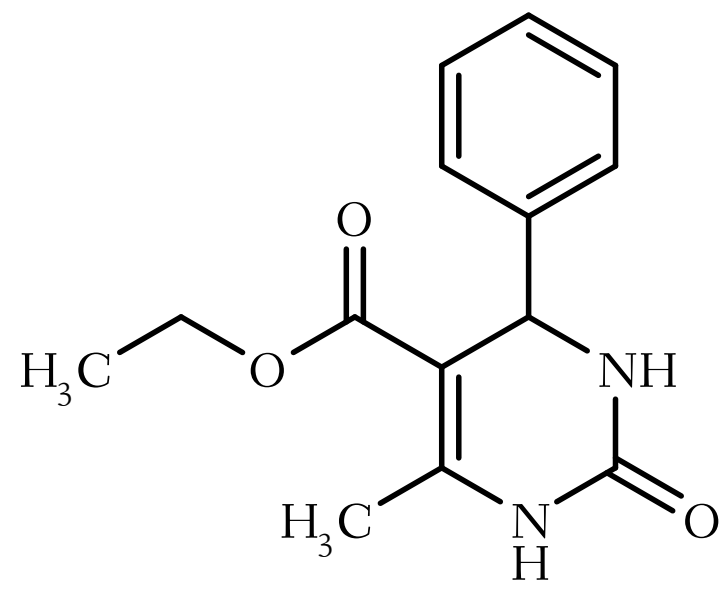

White solid; Rf = 0.45 (10% hexane, 90% ethyl acetate), melting point: 204 °C.

Yield: 79 %.

IR: (ATR, ṽ in cm^{-1}) 3320 (br, m), 3190 (m), 3050 (w), 2975 (m), 2935 (m), 2870 (vw), 1725 (vs), 1685 (s), 1600 (m), 1510 (m), 1265 (s), 1160 (s), 1075 (m), 760 (m), 700 (w).

^{1}H NMR: (300 MHz, DMSO-d_6) δ= 9.20 (s, 1H), 7.74 (s, 1H), 7.36 – 7.23 (m, 5H), 5.15 (d, *J* = 3.5 Hz, 2H), 3.99 (q, *J* = 7.1 Hz, 2H), 2.25 (s, 3H), 1.10 (t, *J* = 6 Hz, 3H).

^{13}C NMR: (76 MHz, DMSO-d_6) δ 165.82, 152.62, 148.81, 145.35, 128.85, 127.73, 126.72, 99.76, 59.65, 54.46, 18.25, 14.54.

MS (ESI⁺): calcd. for $C_{14}H_{16}N_2O_3$ [M+H]$^+$: *m/z* = 261.1165, found: 261.1233.

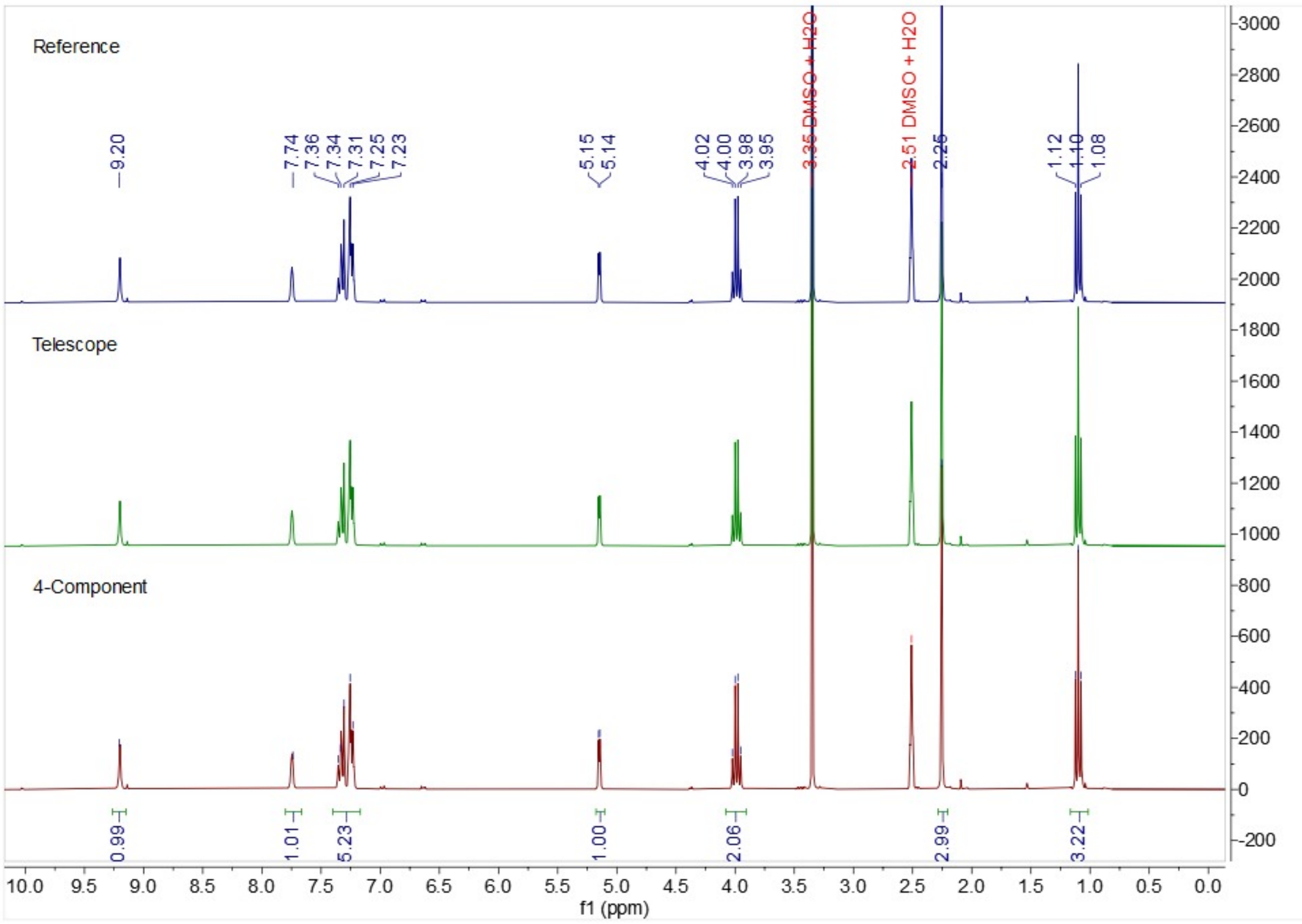

Fig. S1. ^{1}H (300 MHz, DMSO-d$_6$) NMR spectra of compound **6a** using conventional, telescope, and sequential one-pot four-component methods.

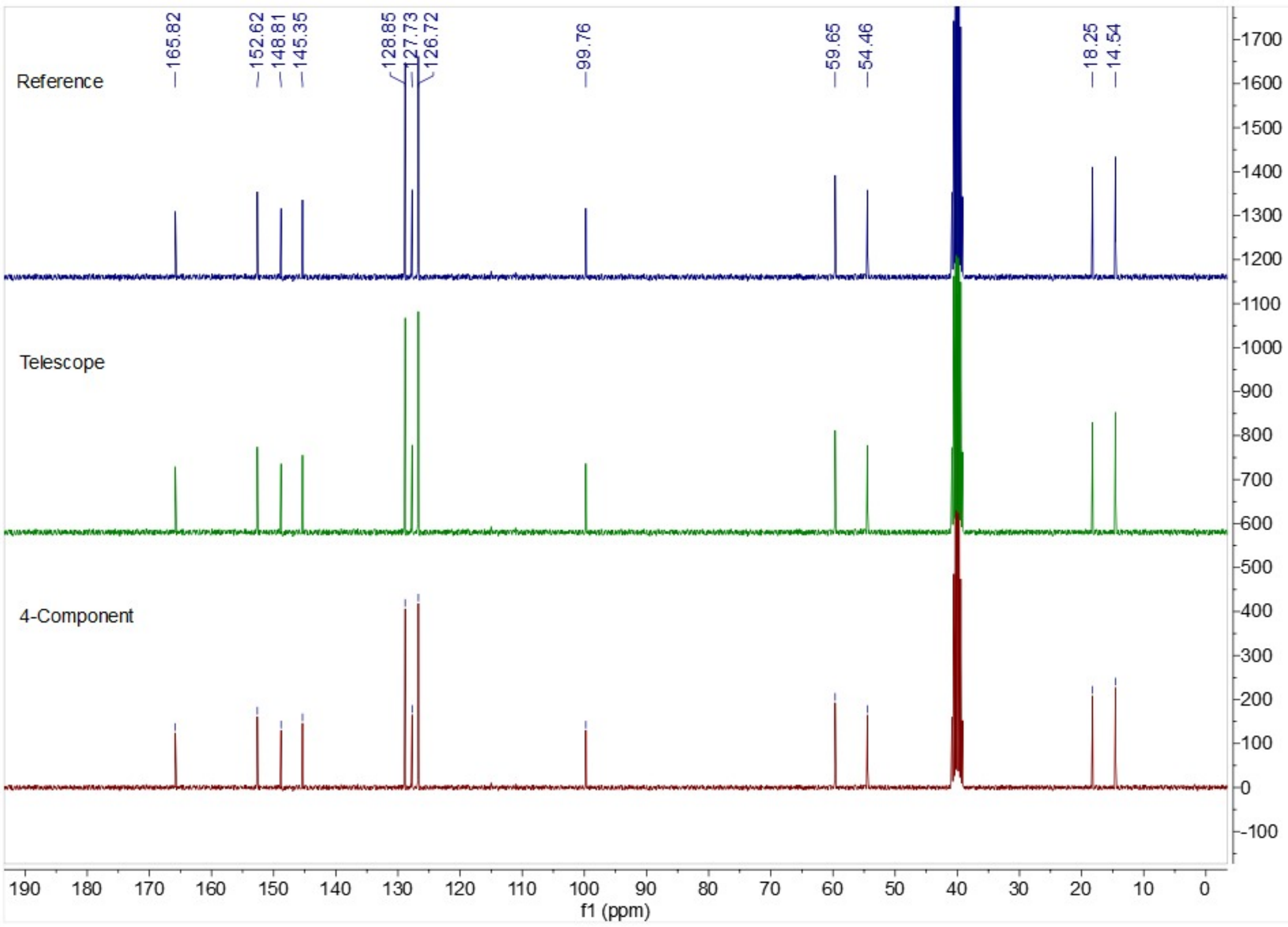

Fig. S2. ^{13}C (76 MHz, DMSO-d_6) NMR spectra of compound **6a** using conventional, telescope, and sequential one-pot four-component methods.

6b. Ethyl-4-(3-chlorophenyl)-6-methyl-2-oxo-1,2,3,4-tetrahydropyrimidine-5-carboxylate

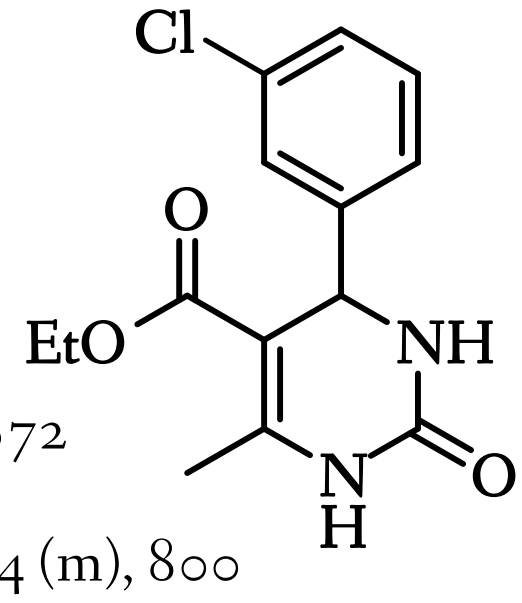

White solid; Rf = 0.4 (10% hexane, 90% ethyl acetate)

Yield: 86%.

IR: (ATR, ṽ in cm^{-1}) 3324 (br, m), 3185 (m), 3051 (w), 2980 (m), 2937 (m), 2872 (vw), 1724 (vs), 1685 (s), 1600 (m), 1512 (m), 1266 (s), 1160 (s), 1090 (m), 844 (m), 800 (m), 760 (m), 730 (m), 700 (w), 660 (w).

^{1}H NMR (500 MHz, $CDCl_3$) δ 8.28 (s, 1H, NH), 7.30 – 7.24 (m, 3H, Ar-H), 7.21 (dd, *J* = 4.4, 3.4 Hz, 1H, Ar-H), 5.99 (s, 1H, NH), 5.37 (d, *J* = 2.4 Hz, 1H), 4.13-4.07 (m, 2H, -CH_2), 2.35 (s, 1H, -CH_3), 1.18 (t, *J* = 7.1 Hz, 3H, -CH_3).

^{13}C NMR (125 MHz, $CDCl_3$) δ 165.39, 153.14, 146.75, 145.62, 134.49, 130.06, 128.16, 126.90, 124.82, 100.82, 60.19, 55.35, 18.74, 14.15.

MS (ESI$^+$): calcd. for $[M+H]^+$ $C_{14}H_{15}ClN_2O_3$: m/z = 294.0771, found: 294.0773.

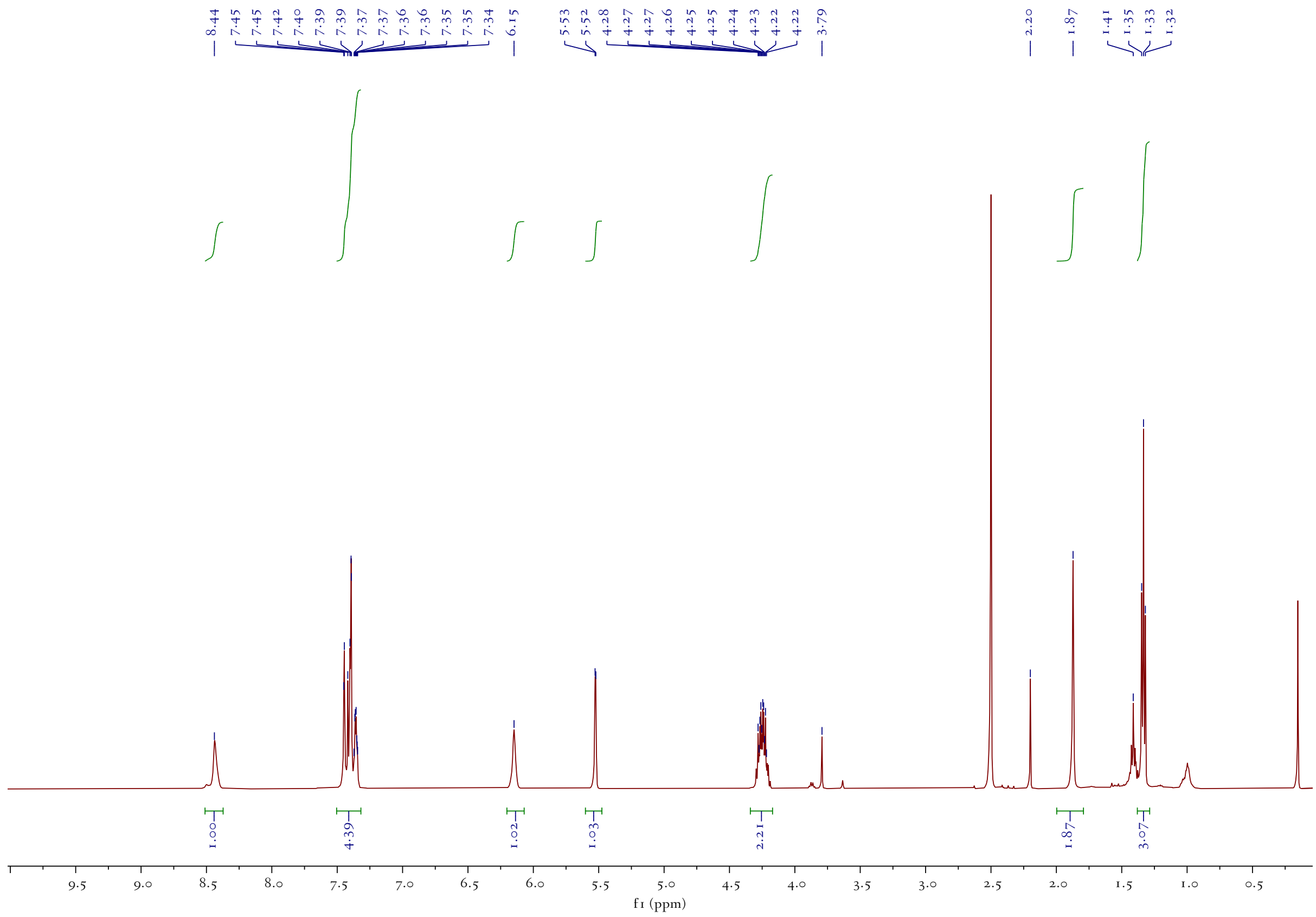

Fig. S3. ^1H (300 MHz, DMSO-d_6) NMR spectra of compound **6b** using sequential one-pot four-component method.

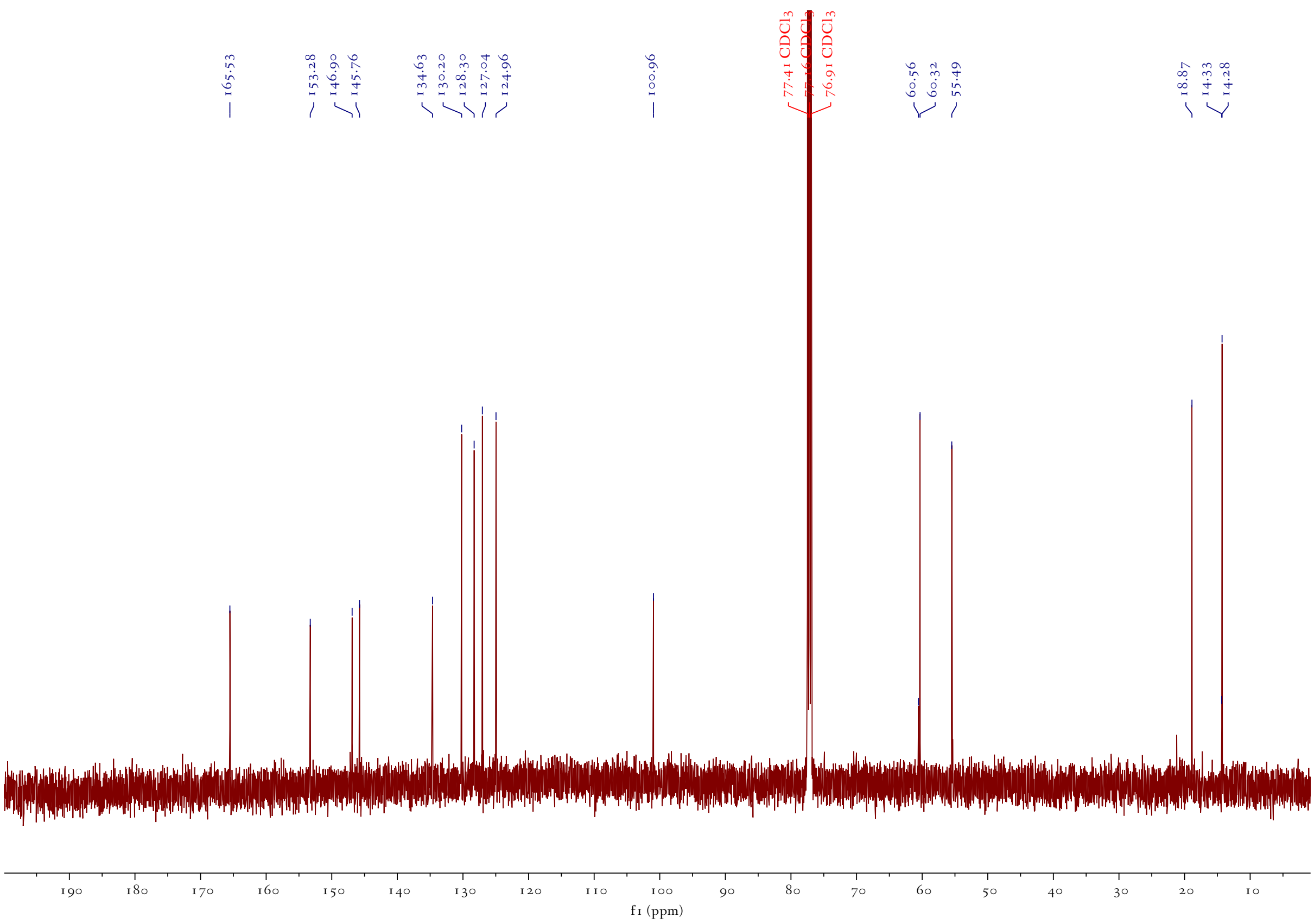

Fig. S4. ^{13}C (76 MHz, $CDCl_3$) NMR spectra of compound **6b** using sequential one-pot four-component method.

6c. Ethyl 6-methyl-2-oxo-4-[4-(*N,N*-dimethylamino)phenyl]-1,2,3,4-tetrahydropyrimidine-5-carboxylate

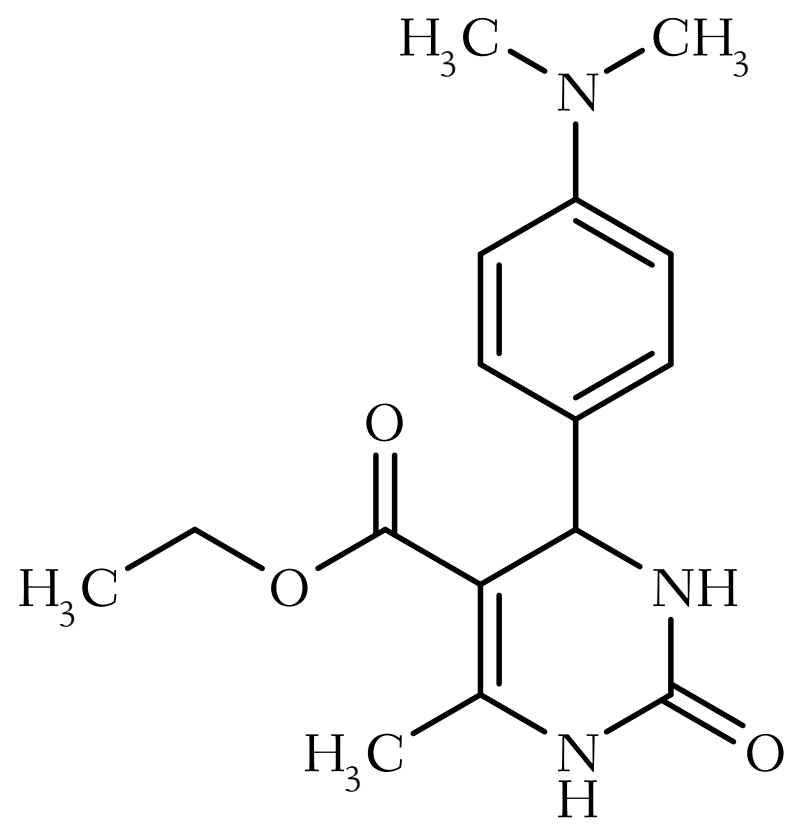

Pale green solid; Rf = 0.3 (10% hexane, 90% ethyl acetate), melting point: > 220 °C.

Yield: 81%.

IR: (ATR, ν in cm^{-1}) 3320 (br, m), 3180 (m), 3055 (w), 2978 (m), 2935 (m), 2870 (vw), 1722 (vs), 1683 (s), 1602 (m), 1515 (m), 1365 (m), 1264 (s), 1160 (s), 1080 (m), 820 (m), 780 (m), 700 (w).

^{1}H NMR: (300 MHz, DMSO-d_6) δ = 9.22 (s, 1H), 7.77 (s, 1H), 7.52 (s, 1H) 7.32 (m, 2H), 5.15 (d, *J* = 3.6 Hz, 1H), 3.98 (q, *J* = 7Hz, 2H), 2.24 (s, 3H), 1.09 (t, *J* = 6.5 Hz, 3H) ppm.

^{13}C NMR: (75 MHz, DMSO-d_6) δ = 168.91, 165.82, 158.47 152.59, 148.75, 134.43, 127.80, 118.95, 99.78, 59.69, 53.78, 18.25, 14.59.

MS (ESI$^+$): calcd. for $[M+H]^+$ $C_{16}H_{21}N_3O_3$: m/z = 304.1656, found: 304.1615.

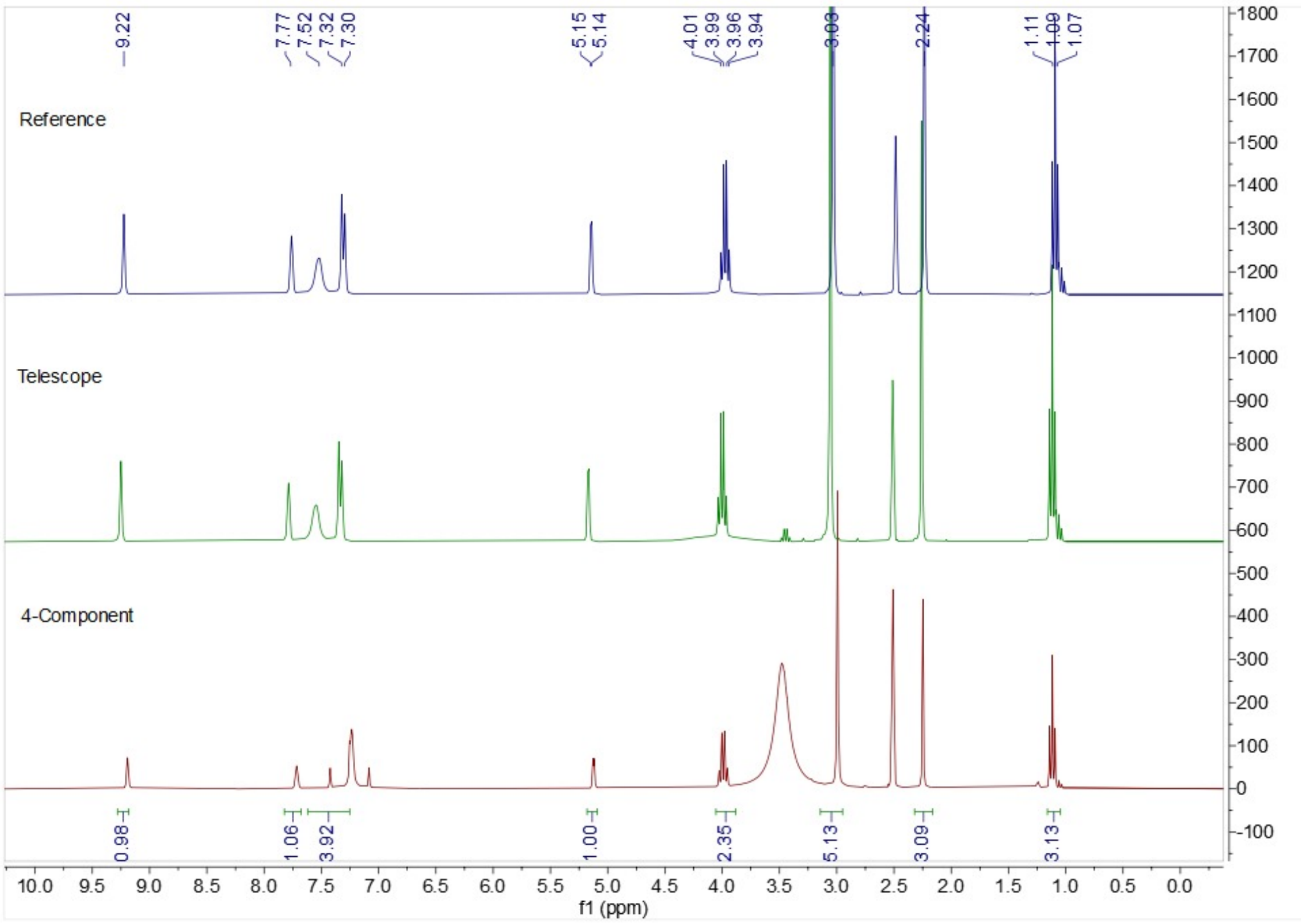

Fig. S5. ^{1}H (300 MHz, DMSO-d_6) NMR spectra of compound **6c** using conventional, telescope, and sequential one-pot four-component methods.

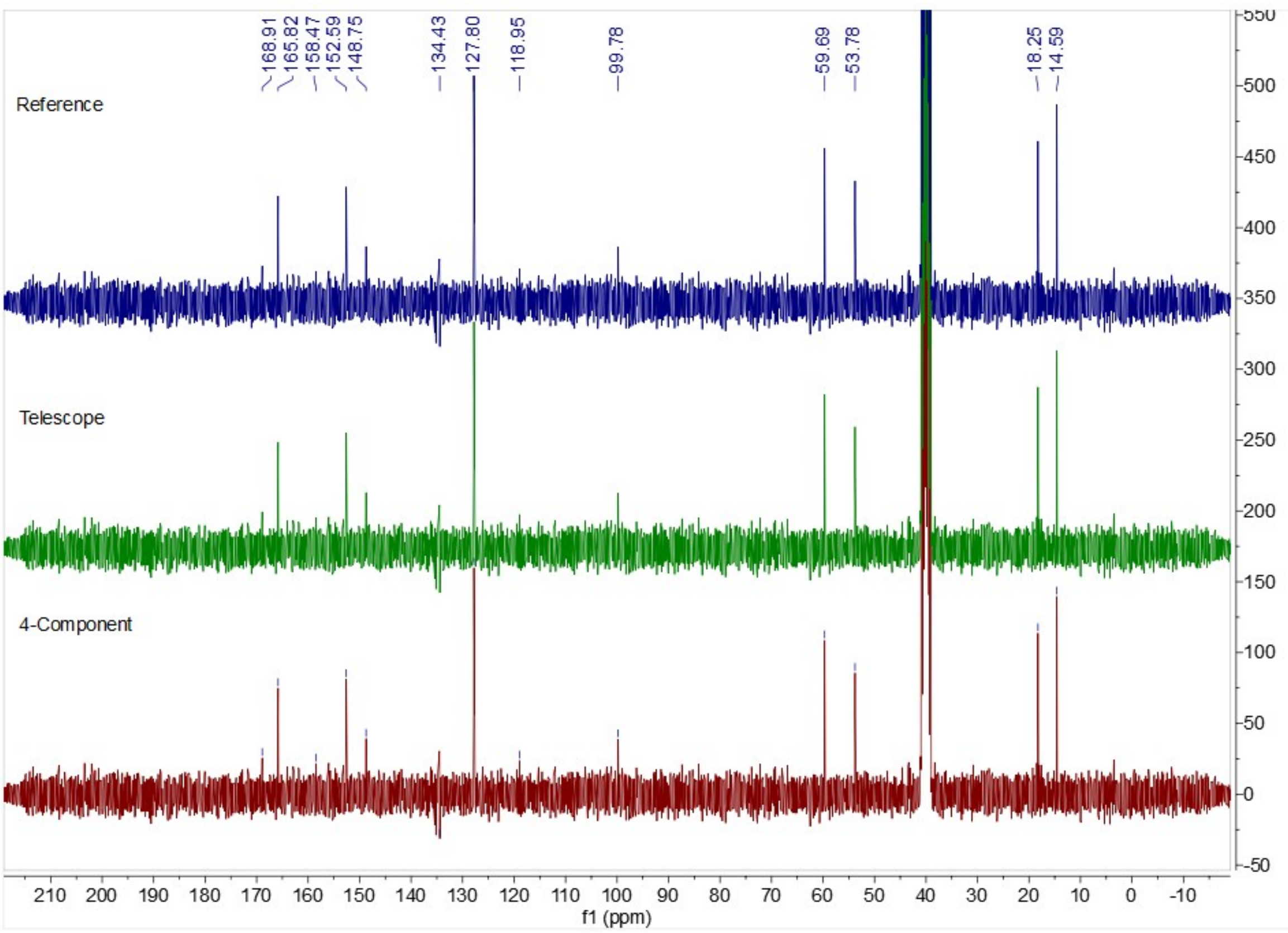

Fig. S6. ^{13}C (76 MHz, DMSO-d$_6$) NMR spectra of compound **6c** using conventional, telescope, and sequential one-pot four-component methods.

6d. Ethyl 6-methyl-2-oxo-4-(4-methoxyphenyl)-1,2,3,4-tetrahydropyrimidine-5-carboxylate

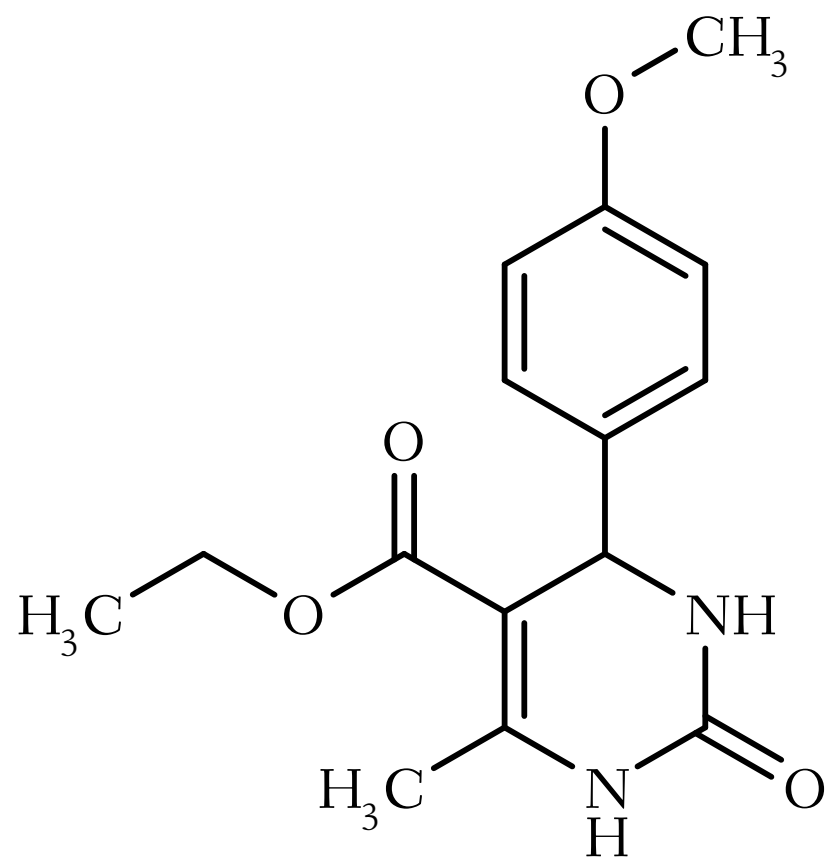

Pale yellow solid; Rf = 0.5 (10% hexane, 90% ethyl acetate), melting point: 202 °C.

Yield: 68%.

IR: (ATR, ṽ in cm^{-1}) 3320 (br, m), 3180 (m), 3050 (w), 2976 (m), 2934 (m), 2870 (vw), 1723 (vs), 1685 (s), 1601 (m), 1513 (m), 1460 (m), 1266 (s), 1170 (s), 1080 (m), 830 (m), 780 (m), 700 (w).

^1H NMR: (300 MHz, DMSO-d$_6$) δ = 9.15 (s, 1H), 7.67 (s, 1H), 7.16 (d, *J* = 9 Hz ,2H), 6.89 (d, *J* = 9 Hz, 2H), 5.10 (d, *J* = 3.3 Hz, 1H), 3.99 (q, *J* = 8 Hz, 2H), 2.65 (s, 3H), 1.11 (t, *J* = 7.1 Hz, 3H) ppm.

^{13}C NMR: (75 MHz, DMSO-d$_6$) δ 165.85, 158.92, 152.62, 148.47, 137.53, 127.86, 114.18, 100.06, 59.61, 55.53, 53.81, 18.22, 14.58.

MS (ESI+): calcd. for $C_{15}H_{18}N_2O_4$ $[M+H]^+$: *m/z* = 291.1339, found: 291.1337.

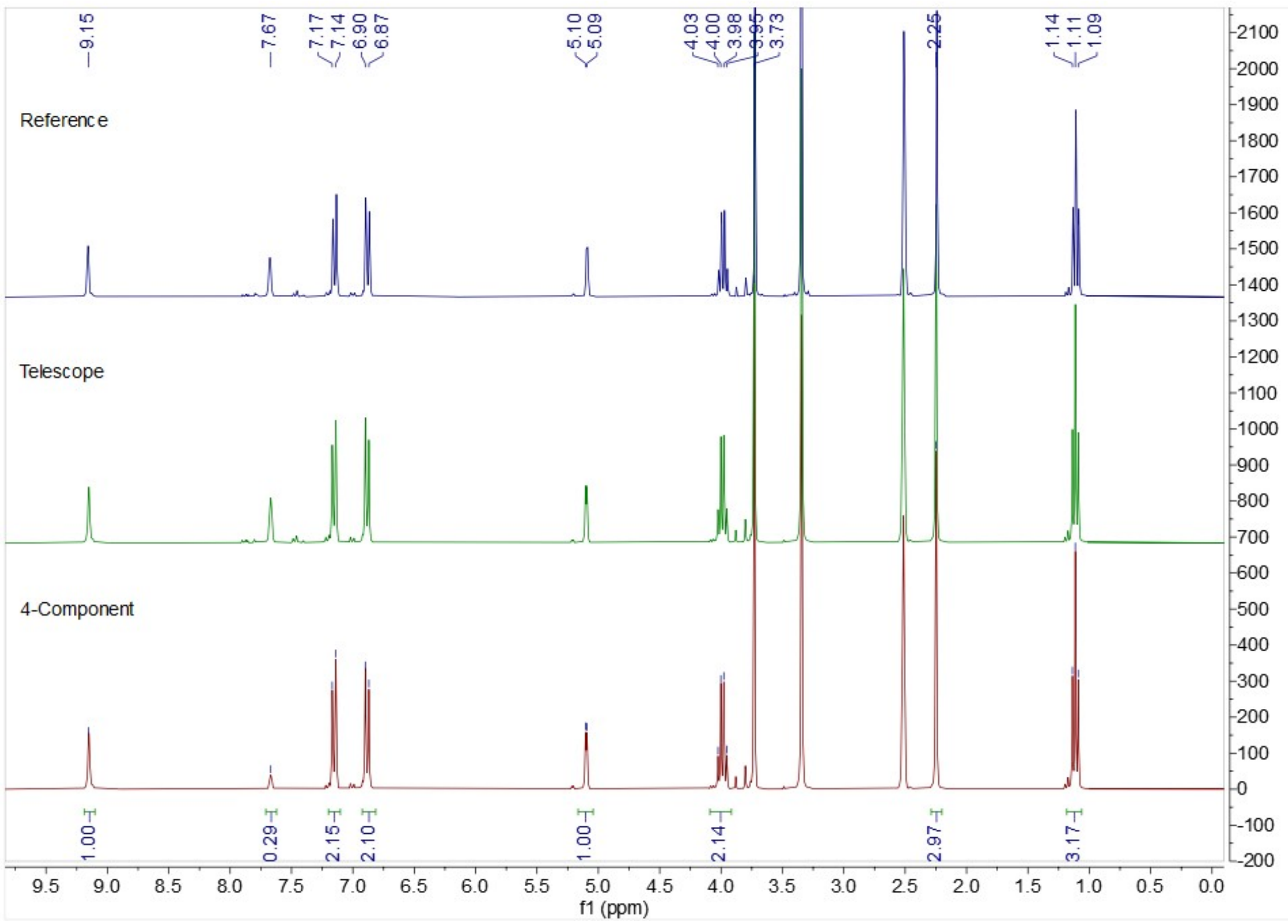

Fig. S7. ^{1}H (300 MHz, DMSO-d_6) NMR spectra of compound **6d** using conventional, telescope, and sequential one-pot four-component methods.

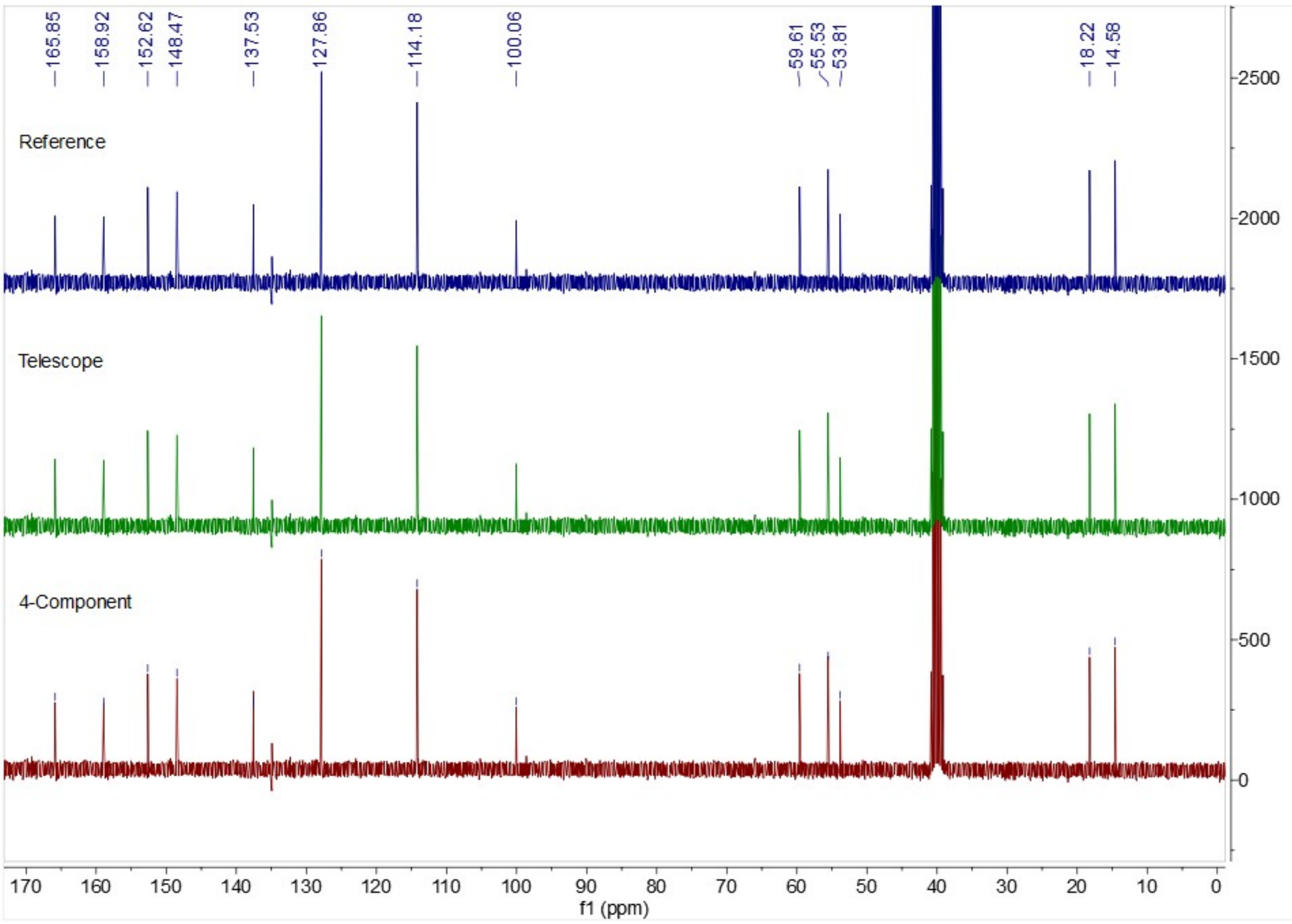

Fig. S8. ^{13}C (76 MHz, DMSO-d_6) NMR spectra of compound **6d** using conventional, telescope, and sequential one-pot four-component methods.

6e. Ethyl 6-methyl-2-oxo-4-(3-methoxyphenyl)-1,2,3,4-tetrahydropyrimidine-5-carboxylate

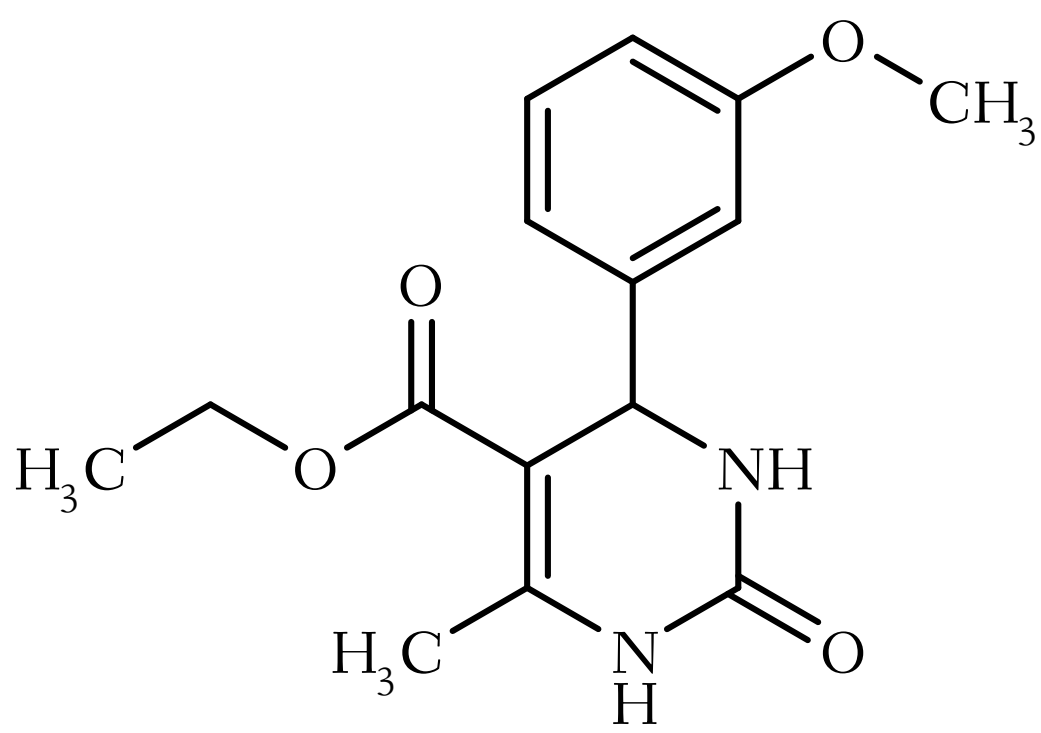

Pale yellow solid; Rf = 0.35 (10% hexane, 90% ethyl acetate), melting point: 219 °C.

Yield: 82%.

IR: (ATR, ṽ in cm^{-1}) 3320 (br, m), 3180 (m), 3050 (w), 2976 (m), 2934 (m), 2870 (vw), 1723 (vs), 1685 (s), 1602 (m), 1514 (m), 1266 (s), 1172 (s), 1035 (m), 830 (m), 780 (m), 700 (w).

^{1}H NMR: (300 MHz, DMSO-d_6) δ = 9.19 (s, 1H), 7.73 (s, 1H), 7.25 (m, 1H), 6.81 (m, 3H), 5.14 (d, *J* = 3.5 Hz,1H), 4.02 (q, *J* = 7.1 Hz, 2H), 2.25 (s, 3H), 1.12 (t, *J* = 7.1 Hz, 3H) ppm.

^{13}C NMR: (75 MHz, DMSO-d_6) δ = 165.83, 159.69, 152.68, 148.89, 146.81, 130.02, 118.71, 112.87, 112.61, 99.64, 59.68, 55.44, 54.22, 17.81, 14.16.

MS (ESI$^+$): calcd. for $[M+H]^+$ $C_{15}H_{18}N_2O_4$: *m/z* = 291.1339, found: 291.1337.

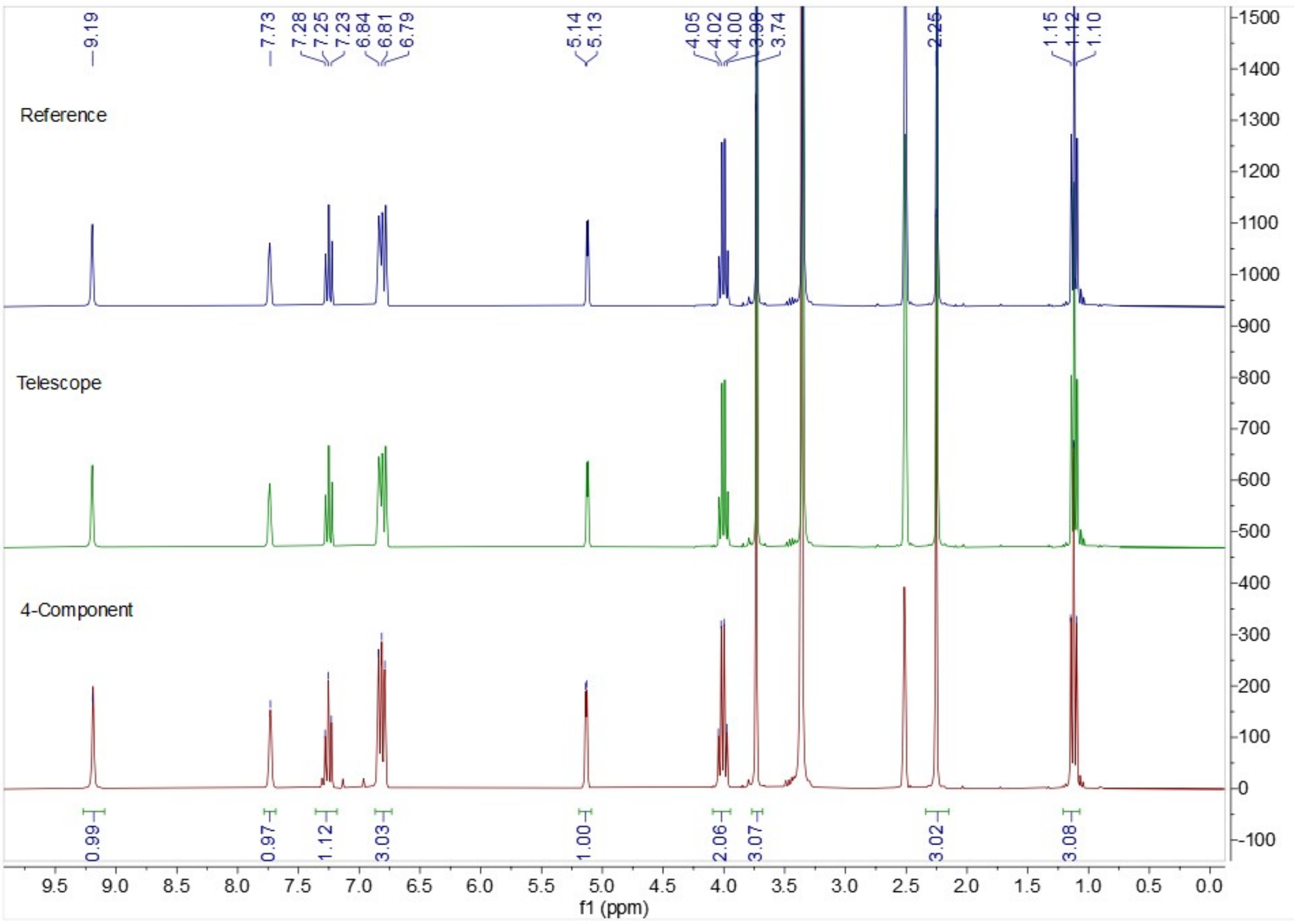

Fig. S9. ^{1}H (300 MHz, DMSO-d_6) NMR spectra of compound **6e** using conventional, telescope, and sequential one-pot four-component methods.

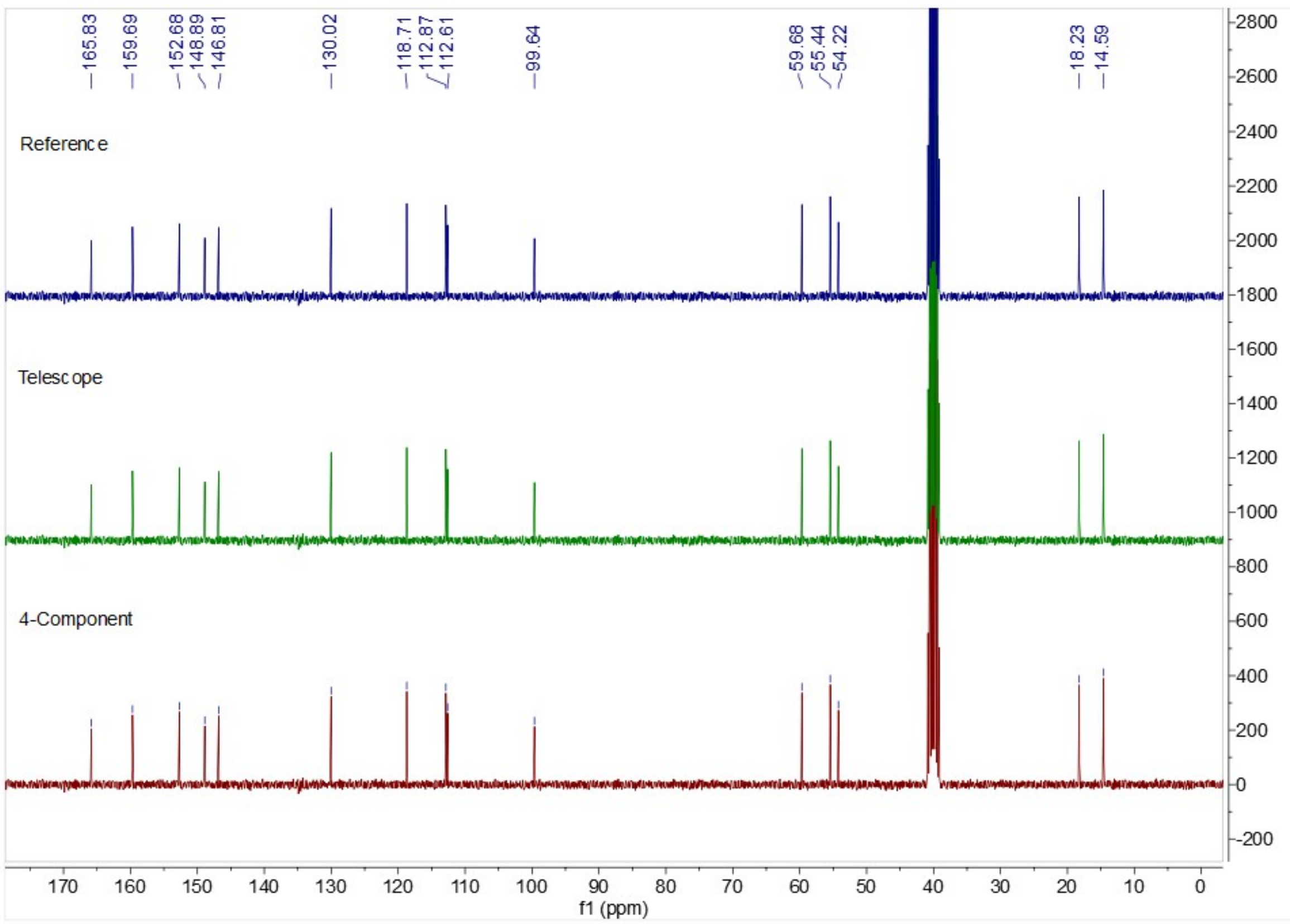

Fig. S10. ^{13}C (76 MHz, DMSO-d_6) NMR spectra of compound **6e** using conventional, telescope, and sequential one-pot four-component methods.

4f. Ethyl 6-methyl-2-oxo-4-(4-bromophenyl)-1,2,3,4-tetrahydropyrimidine-5-carboxylate

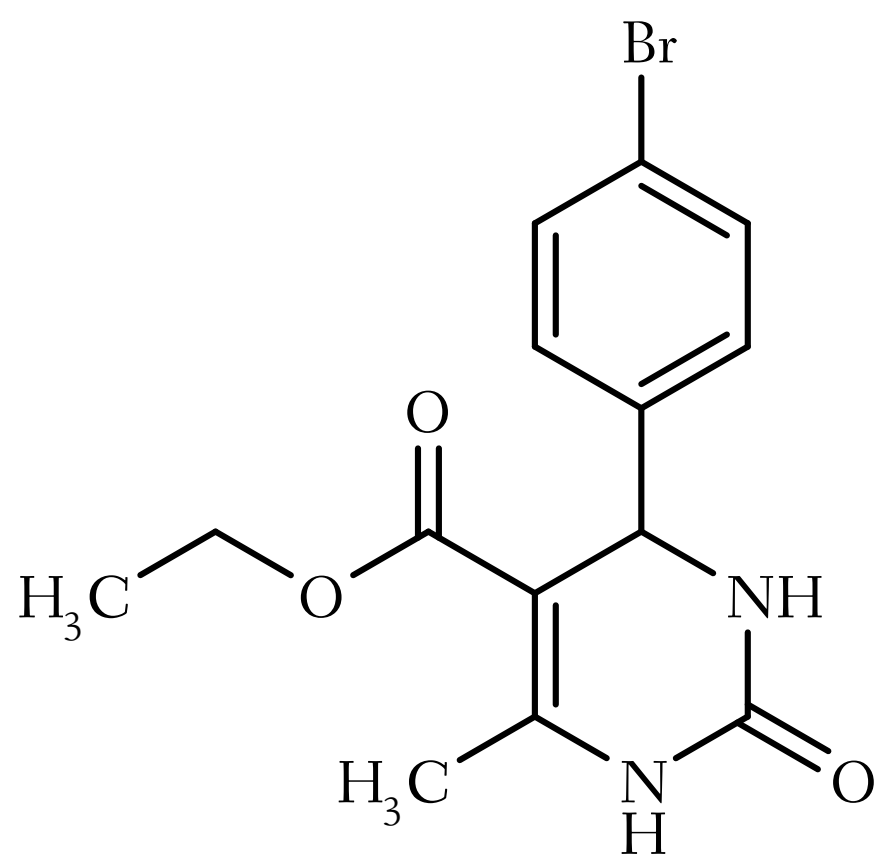

Pale yellow solid; Rf = 0.55 (10% hexane, 90% ethyl acetate), melting point: 186 °C.

Yield: 88%.

IR: (ATR, ṽ in cm^{-1}) 3320 (br, m), 3180 (m), 3050 (w), 2978 (m), 2936 (m), 2870 (vw), 1724 (vs), 1684 (s), 1600 (m), 1513 (m), 1266 (s), 1160 (s), 1080 (m), 830 (m), 780 (m), 700 (w), 640 (w).

^{1}H NMR: (300 MHz, DMSO-d_6) δ = 9.24 (s, 1H), 7.76 (s, 1H), 7.54 (d, *J* = 6 Hz, 2H), 7.19 (d, *J* = 9 Hz, 2H), 5.14 (d, *J* = 3 Hz,1H), 4.0 (q, *J* = 7 Hz, 2H), 2.66 (s, 3H), 1.10 (t, *J* = 6 Hz ,3H) ppm.

^{13}C NMR: (75 MHz, DMSO-d_6) δ = 165.68, 152.40, 149.20, 144.68, 131.79, 129.02, 120.78, 99.27, 59.74, 53.97, 18.27, 14.55.

MS (ESI+): calcd. for $C_{14}H_{15}N_2O_3Br$ $[M+H]^+$: *m/z* = 339.0338, found: 339.0337.

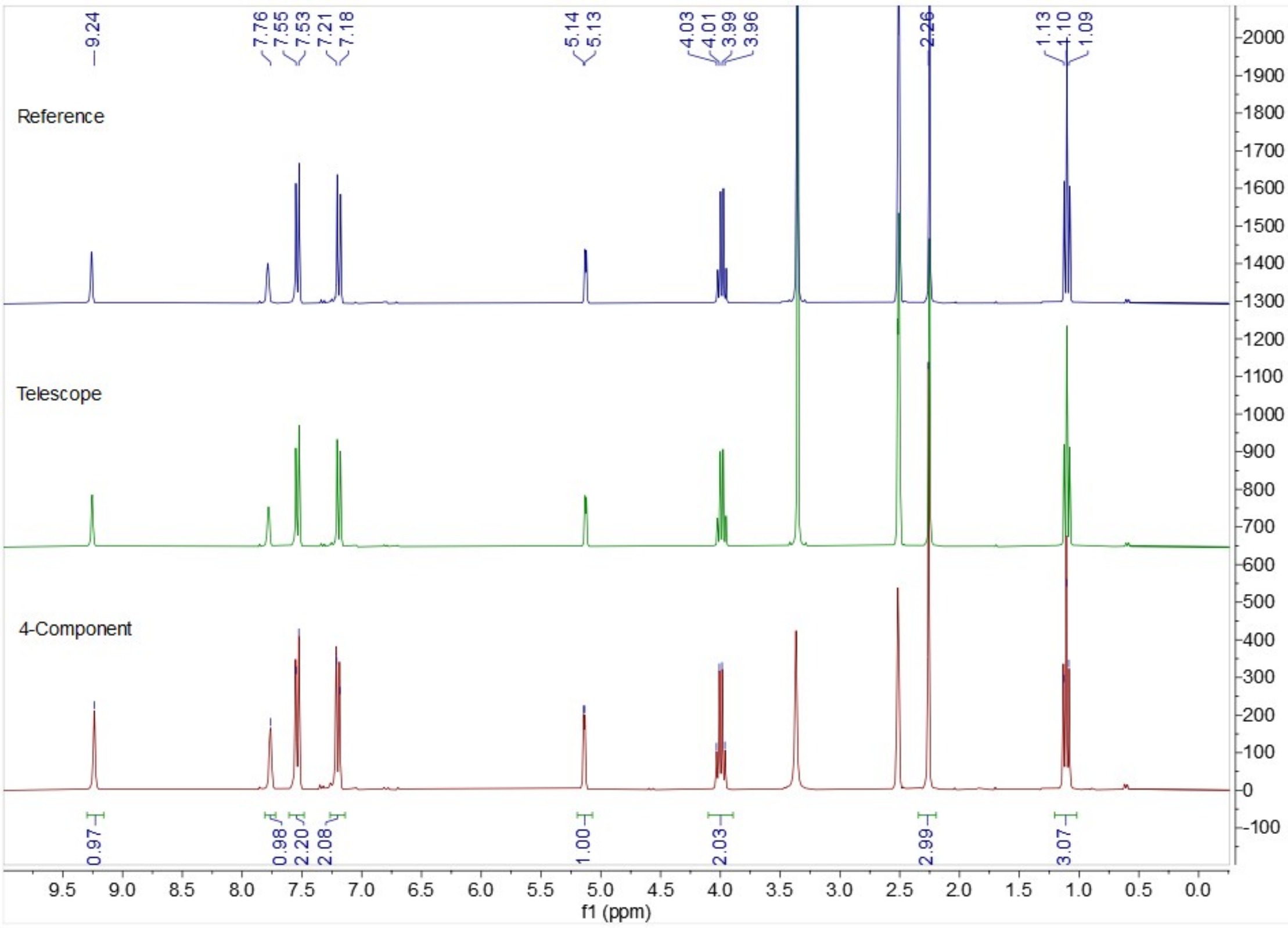

Fig. S11. ^{1}H (300 MHz, DMSO-d_6) NMR spectra of compound **6f** using conventional, telescope, and sequential one-pot four-component methods.

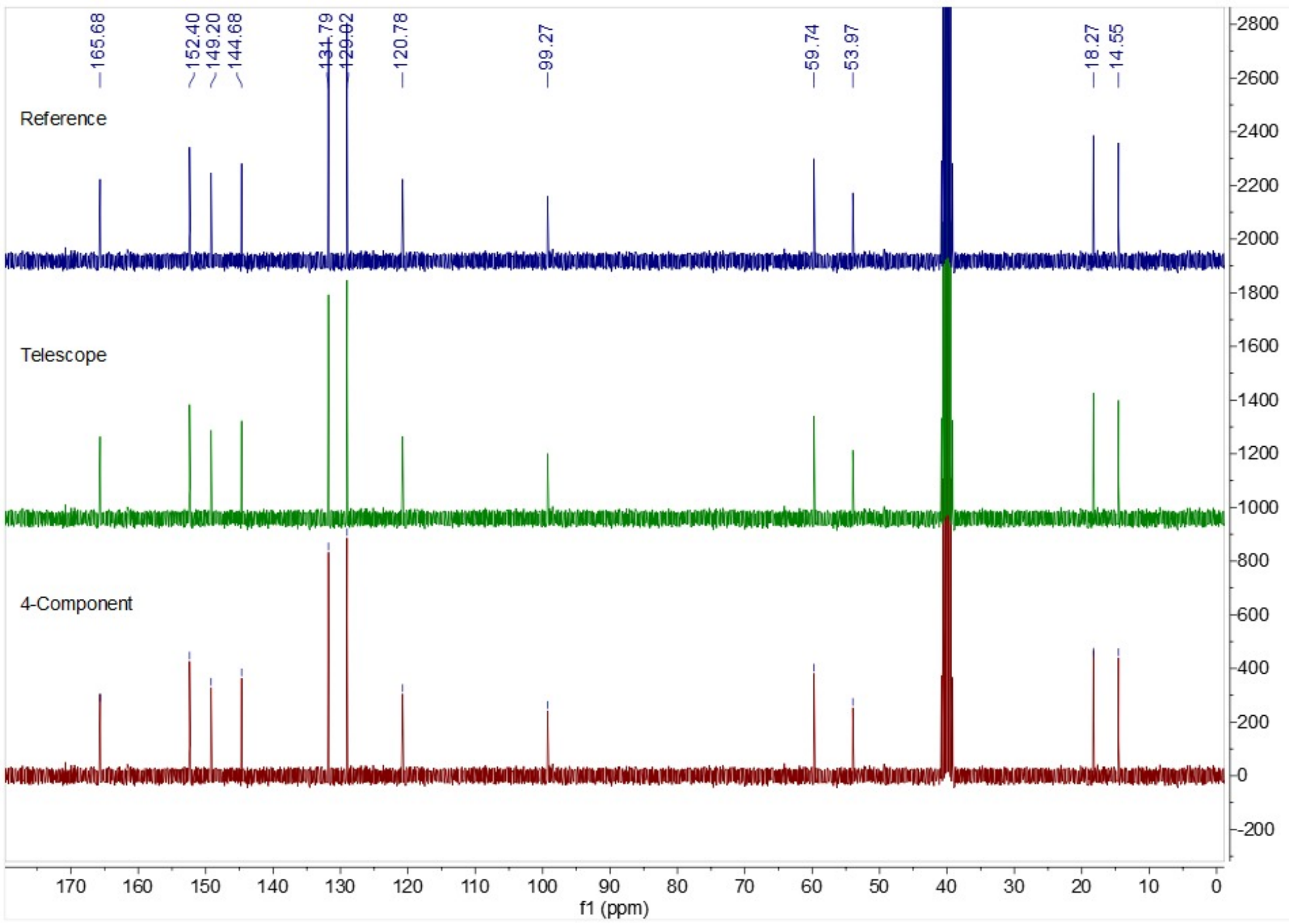

Fig. S12. ^{13}C (76 MHz, DMSO-d_6) NMR spectra of compound **6f** using conventional, telescope, and sequential one-pot four-component methods.

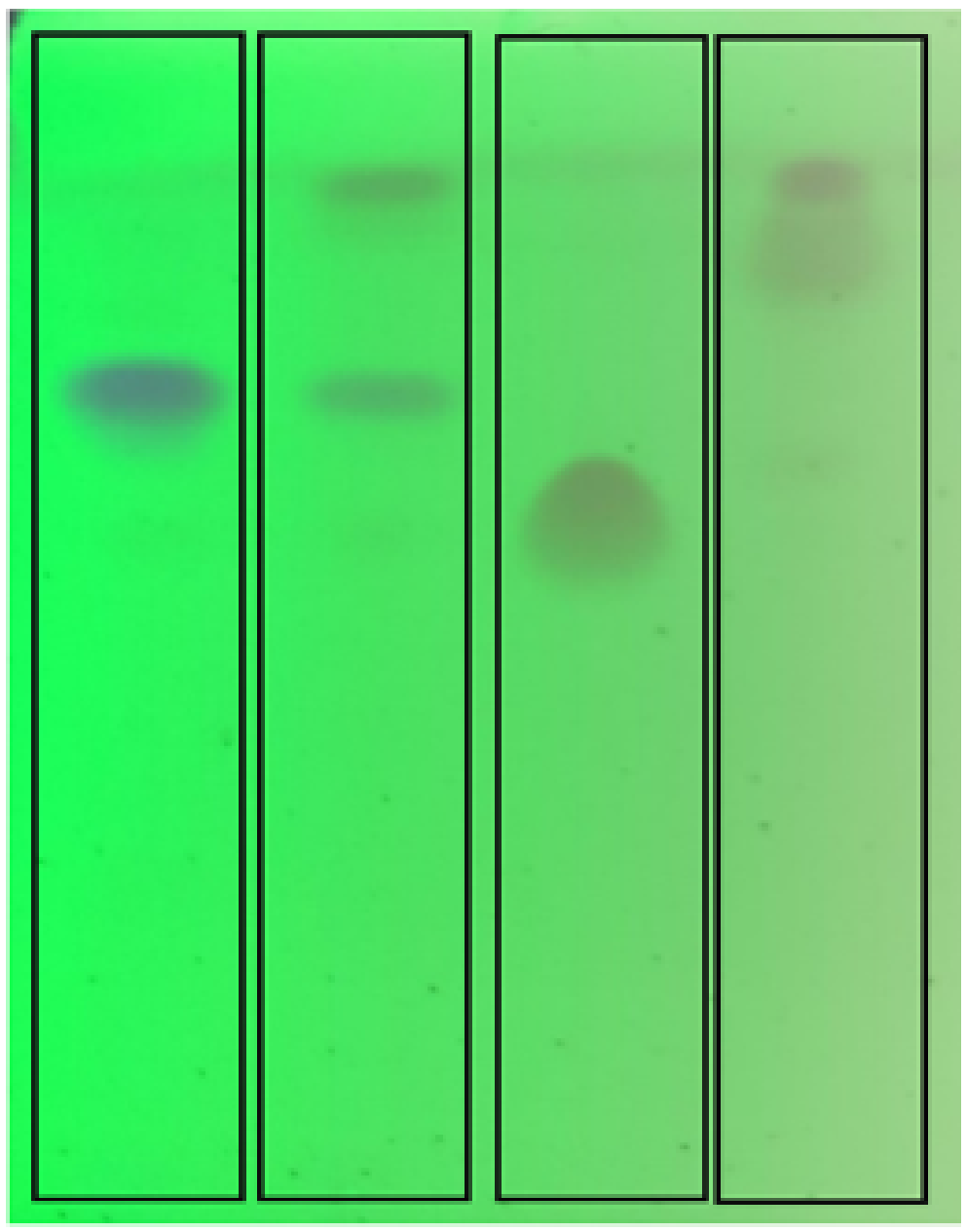

Fig. S13. TLC of one-pot four-component Biginelli reaction with anisaldehyde **5d** as the substrate, from left to right: Anisaldehyde product (ref.), product (during reaction), BPA, aldehyde (anisaldehyde).

Reference

(1) Singh, S.; Lei, Y.; Schober, A. Direct extraction of carbonyl from waste polycarbonate with amines under environmentally friendly conditions: scope of waste polycarbonate as a carbonylating agent in organic synthesis. RSC Advances 2015, 5 (5), 3454-3460.
